# Effect of plastic composition in the combustion material on the Persistent Organic Pollutant content in smoked chicken meat

**DOI:** 10.1371/journal.pone.0350345

**Published:** 2026-06-03

**Authors:** Giang Do Hoang, Uyen Nguyen Thu, Luyen Nguyen Thi, Duong Hoang Thuy, Le Bui Thi Nhat, Nam Vu Duc, Xuyen Nguyen Thi, Thuy Le Minh, Tuan Anh Hoang Le, Tung Nguyen Ngoc, Dat Nguyen Tien

**Affiliations:** 1 Center for High Technology Research and Development, Vietnam Academy of Science and Technology, Hanoi, Vietnam; 2 University of Science and Technology of Hanoi, Vietnam Academy of Science and Technology, Hanoi, Vietnam; VIT University, INDIA

## Abstract

Traditional food smoking in Vietnam often uses biomass fuels, but the incidental inclusion of plastics can generate toxic pollutants. This study evaluated the effects of polyethylene (PE), polystyrene (PS), and polyvinyl chloride (PVC) compared with clean wood on persistent organic pollutants (POPs) in smoked chicken, including PCBs, PCDD/Fs, and PAHs. Samples were smoked under controlled conditions, analyzed by GC–MS/MS, and assessed with univariate and multivariate statistics. Clean wood yielded low contamination, while PE introduced lighter PAHs and dioxins, PS was associated with mid-weight PAHs and specific PCBs (66, 195), and PVC caused the most severe contamination with 12 PCDD/Fs, 21 PCBs (e.g., 189, 101, 180, 153), and multiple carcinogenic PAHs (e.g., benzo[a]pyrene). Statistical tests confirmed significant differences among fuel groups (p < 0.01). The contamination gradient (W < PE < PS < PVC) highlights the urgent need to prohibit plastic-contaminated fuels in traditional smoking to safeguard food safety and public health.

## Introduction

The smoking method has been applied for centuries in traditional food processing, serving not only as a means of preservation but also as a way to impart distinctive sensory qualities to food. This process typically involves burning natural biomass such as wood, straw, or agricultural residues, producing smoke rich in antibacterial and antioxidant compounds, as well as flavor-enhancing agents [1]. In Vietnam, smoking is a long-standing culinary practice used for buffalo, chicken, and pork, particularly in rural kitchens. Traditionally, fuels include paper, wood, or straw; however, in practice, packaging materials or waste wood coated with paint are sometimes used as substitutes due to convenience or cost. Such materials often contain plastics, paints, or adhesives, which release hazardous by-products upon combustion. When fuel sources include plastic components, the combustion process generates a wide spectrum of toxic compounds, notably dioxin-related compounds (DRCs), including polychlorinated dibenzo-p-dioxins (PCDDs), polychlorinated dibenzofurans (PCDFs), and dioxin-like polychlorinated biphenyls (dl-PCBs) [2]. These compounds are typically formed during incomplete combustion of chlorine-containing substances at elevated temperatures, especially in the presence of synthetic polymers such as polyvinyl chloride (PVC) [3]. Numerous studies have confirmed that burning plastic-contaminated fuels elevates the levels of PCBs, dioxins, and furans in smoke, which subsequently deposit onto and penetrate food matrices [1,3,4]. The presence of these contaminants in smoked foods poses serious risks to food quality and human health. The World Health Organization (WHO) classifies dioxins, furans, and PCBs as Group 1 carcinogens [5]. They are persistent organic pollutants (POPs), highly stable in the environment and capable of bioaccumulating in fatty tissues [6]. Chronic dietary exposure has been linked to endocrine disruption, immunotoxicity, reproductive and developmental impairments, as well as elevated risks of cancers, particularly hepatocellular carcinoma and lung cancer [7,8]. Moreover, these compounds adversely affect the nervous system, impairing cognitive development in children and increasing vulnerability during pregnancy [9]. Long-term exposure has also been associated with metabolic and chronic diseases, including type 2 diabetes, cardiovascular disorders, and autoimmune syndromes [9,10].

Despite these findings, important knowledge gaps remain. Most previous studies have focused on individual pollutant groups or specific fuel types, and systematic comparisons across different plastic materials under controlled conditions are still limited. In particular, there is a lack of studies that simultaneously evaluate multiple classes of persistent organic pollutants (PCBs, PCDD/Fs, and PAHs) and characterize their congener-level fingerprints in relation to fuel composition. Although a related study from our group examined smoked pork under comparable combustion scenarios [11], the present work is based on an independent smoked chicken experiment with separately generated samples, measurements, and analyses. No data, figures, or tables are duplicated from any previous publication.

Therefore, this study aims to: (i) quantify the concentrations of PCBs, PCDD/Fs, and PAHs in smoked chicken using different fuel types (wood, wood + PE, wood + PS, and wood + PVC); (ii) compare congener profiles and identify characteristic marker compounds associated with each polymer type; and (iii) apply multivariate analyses to discriminate contamination patterns and fingerprints related to fuel composition. The findings highlight the critical importance of monitoring and controlling fuel sources in traditional smoking practices.

## Materials and methods

### Samples and smoking procedures

Nine whole chickens were purchased from local markets, rinsed thoroughly with tap water, and drained. Each chicken was divided into five equal portions (approximately 300 g each). Four portions were assigned to smoking treatments, while the remaining portion served as an unsmoked control and was oven-dried at 90 °C to constant weight (baseline sample). These control samples were subjected to the same analytical procedures as the smoked samples, and target contaminants were not detected (below the method detection limits).

Smoking experiments were conducted using a custom-built box-type smoking chamber (1200 × 600 × 1200 mm, L × W × H) equipped with a separate combustion compartment and an adjustable airflow system. The distance between the combustion source and the sample chamber was maintained at approximately 120 cm to prevent direct flame contact and to ensure that the smoke entering the chamber was sufficiently cooled. The chamber temperature was monitored and maintained within the range of 80–90 °C throughout the smoking process. Relative humidity was not controlled and followed ambient laboratory conditions.

The fuel materials consisted of (i) pure wood (Group W), (ii) wood mixed with polyethylene (PE), (iii) wood mixed with polyvinyl chloride (PVC), and (iv) wood mixed with polystyrene (PS), each at a ratio of 99:1 (w/w). The wood used in all treatments was *Manglietia conifera*, which was washed with water to remove surface impurities and air-dried prior to use. To avoid cross-contamination between treatments, each fuel type was combusted in separate smoking sessions. After each session, the chamber was thoroughly cleaned and ventilated before the next experiment.

Smoking was performed for five consecutive days, 8 hours per day, under identical operational conditions for all treatments. Portions originating from the same chicken were assigned to different treatments to minimize biological variability. After treatment, all samples (smoked and control) were freeze-dried, ground into fine powder, and stored at −20 °C until chemical analysis.

### Experimental

The samples were processed and analyzed following the same procedure as described in our previous publication [11,12]. Briefly, samples were freeze-dried, homogenized, and fortified with isotopically labelled internal standards prior to analysis. Total lipid content was determined gravimetrically from a portion of the extract. Persistent organic pollutants (PAHs, PCBs, and PCDD/Fs) were extracted using accelerated solvent extraction, followed by solvent exchange, lipid removal, and multi-step clean-up and enrichment procedures tailored to each compound class. Quantitative determination was carried out by GC–MS/MS for PAHs and PCBs and by high-resolution GC–MS for PCDD/Fs using isotope-dilution calibration. Instrumental data were processed using Xcalibur 4.2 (Thermo Scientific, USA), and statistical analyses were performed in R (R Foundation for Statistical Computing, Vienna, Austria). The analytical procedures applied in this study were validated using standard quality assurance and quality control criteria, as described in the Supplemental Materials. Briefly, method performance was evaluated in terms of linearity, sensitivity, and accuracy using multi-level calibration with native and isotopically labelled standards. Method detection limits (MDLs) and method quantification limits (MQLs) were calculated according to US EPA guidelines and reported on a lipid-weight basis. Extraction efficiency and overall method accuracy were assessed through recovery experiments employing ^13^C-labelled internal standards spiked prior to extraction. Acceptance of analytical results was based on established EPA recovery and performance criteria. All analytes met EPA acceptance criteria for linearity, sensitivity, and recovery, confirming the robustness of the analytical approach. Detailed validation data, including calibration statistics, MDL/MQL values, and recovery ranges for PAHs, PCBs, and PCDD/Fs, are provided in the Supplemental section.

### Data processing and statistical methods

Instrumental data were processed using Xcalibur version 4.2 (Thermo Scientific, USA), and statistical analyses were performed in R (R Foundation for Statistical Computing, Vienna, Austria). Non-detected (N.D.) values were replaced with one-half of the corresponding method detection limit (MDL/2) prior to calculation of total concentrations and subsequent multivariate/statistical analyses; detailed procedures are provided in the Supporting Information. Because portions from the same chicken were assigned across treatments, differences among fuel groups were evaluated using the Friedman test for repeated measures, followed by paired Wilcoxon signed-rank post-hoc tests with Benjamini–Hochberg correction for multiple comparisons. Statistical significance was defined at p < 0.05. Prior to multivariate analysis, data were standardized, and principal component analysis (PCA) together with hierarchical cluster analysis (HCA; Euclidean distance, Ward’s method) were applied to visualize contamination patterns and group discrimination.

### Ethics statement

Chicken samples were commercially purchased from local markets, and no live animals were involved in the study. Therefore, formal animal ethics approval was not required.

## Results

### PCDD/Fs and PCBs concentrations in the samples

**Fig 1:**
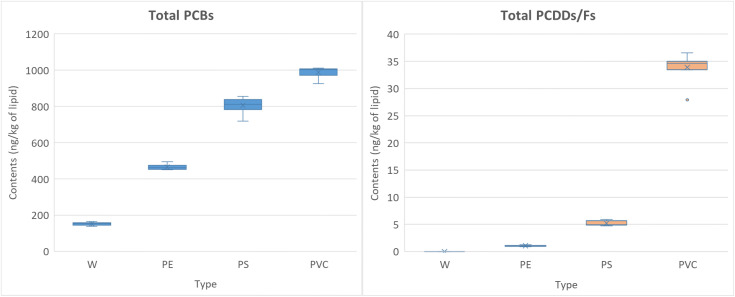
Boxplots of total PCBs (left) and total PCDD/Fs (right) concentrations of the samples. The central line represents the median, the box indicates the interquartile range (IQR), whiskers represent the minimum and maximum values, and “×” denotes the mean.

The analysis of chicken meat smoked with different fuel sources revealed pronounced differences in contamination levels of total PCBs and total PCDDs/Fs (S1 Table, Fig 1). When clean wood (W) was used, the mean total PCB concentration remained low (152 ± 8 ng/kg of lipid), with values ranging from 139 to 165 ng/kg of lipid, and no detectable PCDDs/Fs were observed. In this group, only a limited number of lower-chlorinated PCB congeners were present.

In the PE group, 25 PCB congeners and a single PCDD/F congener were detected. Total PCB levels averaged 465 ± 14 ng/kg of lipid, ranging from 451 to 494 ng/kg of lipid, while total PCDDs/Fs were minimal (1.06 ± 0.11 ng/kg of lipid, range: 0.89–1.23 ng/kg of lipid). This indicates that although PE contains no chlorine, its combustion still promoted PCB formation. The PS group exhibited more pronounced contamination, with nearly all PCB congeners and multiple PCDD/F congeners present. The mean total PCB concentration reached 806 ± 42 ng/kg of lipid (range: 720–856 ng/kg of lipid), and total PCDDs/Fs averaged 5.21 ± 0.44 ng/kg of lipid (range: 4.70–5.88 ng/kg of lipid). Compared to PE, PS combustion yielded both higher levels and a broader spectrum of contaminants, reflecting more complex formation pathways under combustion conditions. The most severe contamination occurred in the PVC group, where all 29 PCB congeners and a wide range of PCDD/F congeners were detected. Total PCBs reached 990 ± 31 ng/kg of lipid (range: 926–1011 ng/kg of lipid), while PCDDs/Fs peaked at 33.90 ± 2.44 ng/kg of lipid (range: 27.9–36.6 ng/kg of lipid). The wider dispersion in PVC samples suggests highly variable but intense formation of toxic compounds, consistent with the chlorine-rich structure of PVC.

**Fig 2:**
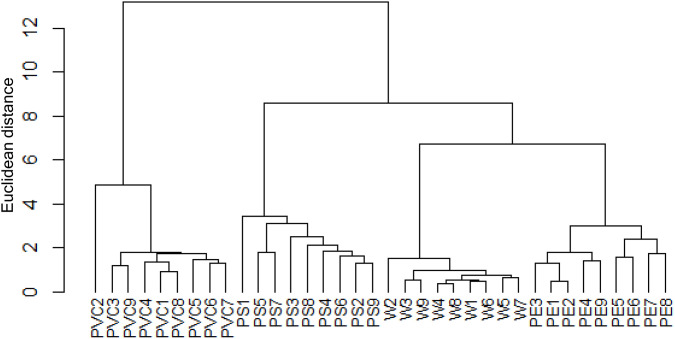
Hierarchical cluster analysis dendrogram of the PCDD/Fs and PCBs in smoked chicken meat, constructed using Euclidean distance and Ward’s method after data standardization.

The hierarchical cluster analysis (HCA) further supported the differentiation of samples according to the type of smoking fuel (Fig 2). The dendrogram clearly separated the PVC group from all others, reflecting its distinct contamination profile with markedly higher levels of both PCBs and PCDDs/Fs. The PS samples formed a separate cluster, positioned between the PVC and the lower-contamination groups, consistent with their intermediate contamination levels. In contrast, the PE and W groups clustered together, although still distinguishable, indicating that PE combustion resulted in elevated PCB concentrations but remained closer in profile to clean wood compared to PS and PVC.

This clustering pattern corroborates the quantitative results: contamination followed the order W <<PE < PS < PVC. The distinct grouping of PVC samples highlights the strong influence of chlorine-rich fuels on dioxin-related compound formation, while the intermediate position of PS indicates that even non-chlorinated plastics can substantially alter contamination profiles. The HCA thus confirms that the type of fuel used in smoking is the primary determinant of both the magnitude and congener distribution of toxic compounds in smoked chicken meat.

**Fig 3:**
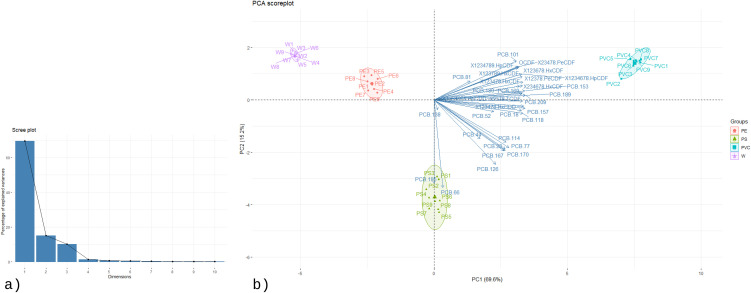
The PCA (a) scree plot and (b) biplot of PCBs and PCDD/Fs concentrations of the samples.

To further explore the relationships among contaminants and identify key compounds driving the separation between groups, principal component analysis (PCA) was performed. The first two principal components explained 84.7% of the total variance (PC1: 69.6%, PC2: 15.2%), indicating that most of the variability in the dataset could be effectively captured in a two-dimensional space. The scree plot (Fig 3a) and eigenvalue distribution confirmed the strong dominance of PC1, suggesting that a small set of pollutants accounted for the majority of group differentiation. The PCA biplot (Fig 3b) showed a clear clustering of samples according to fuel type, consistent with the HCA results. Meat smoked with clean wood (W) was grouped tightly and was positioned near the origin, reflecting low contamination and the absence of detectable PCDDs/Fs. In contrast, the PE group was slightly separated along PC2, mainly due to the appearance of specific dioxin congeners such as 2,3,7,8-TCDF and 1,2,3,7,8-PeCDF, as well as marginal increases in lighter PCBs. Non-parametric statistical testing using the Friedman test, followed by post-hoc pairwise comparisons using the Wilcoxon signed-rank test with Benjamini–Hochberg (BH) correction, confirmed that these congeners were significantly more abundant in PE compared with W (*p* < 0.05). The PS group was clearly distinguished from both W and PE, characterized by a broader contaminant profile including eight PCDD/F congeners and twenty-one PCB congeners. Among them, PCB 66 and PCB 195 showed significantly higher concentrations relative to the other groups (Friedman test with Wilcoxon signed-rank post-hoc and BH correction, *p* < 0.01), suggesting they serve as marker compounds for PS combustion. PVC samples were separated furthest along PC1, highlighting their distinct contamination profile. This group contained the widest spectrum of pollutants, with twelve PCDD/F congeners and twenty-one PCB congeners detected. The concentrations of PCDDs/Fs in PVC were consistently higher than in all other groups (Friedman test, *p* < 0.001). Furthermore, several highly chlorinated PCBs—including PCB 189, PCB 101, PCB 180, and PCB 153—were significantly elevated compared to W, PE, and PS (Wilcoxon signed-rank test with BH correction, *p* < 0.01), making them characteristic markers of chlorine-rich fuel combustion.

In summary, both univariate and multivariate analyses demonstrated that the use of plastic-contaminated fuels in smoking markedly increased the levels and diversity of PCBs and PCDDs/Fs in chicken meat. Clean wood produced negligible contamination, whereas PE and PS combustion generated distinct sets of marker compounds, and PVC resulted in the most severe and complex pollutant profile. These findings highlight the critical role of fuel composition in shaping both the magnitude and fingerprint of dioxin-related contaminants, underscoring the substantial food safety risks associated with burning plastics during traditional smoking practices.

### PAHs concentrations in the samples

In addition to PCBs and PCDDs/Fs, the analysis of polycyclic aromatic hydrocarbons (PAHs) in smoked chicken meat also revealed pronounced differences depending on the type of fuel used (S3 Table). When clean wood (W) was applied, the mean total PAH concentration was relatively low (544 ± 44 μg/kg of lipid), with values ranging from 481 to 617 μg/kg of lipid. In contrast, the use of plastic-contaminated fuels markedly elevated PAH levels. In the PE group, total PAHs averaged 1730 ± 95 μg/kg of lipid (range: 1567–1838 μg/kg of lipid), while the PS group reached slightly higher concentrations at 1825 ± 121 μg/kg of lipid (range: 1670–1997 μg/kg of lipid). Both groups showed more than a threefold increase compared with clean wood. The highest levels were again observed in the PVC group, where total PAHs averaged 2766 ± 158 μg/kg of lipid, with concentrations spanning from 2524 to 2968 μg/kg of lipid. This represents approximately a fivefold increase relative to W samples.

**Fig 4:**
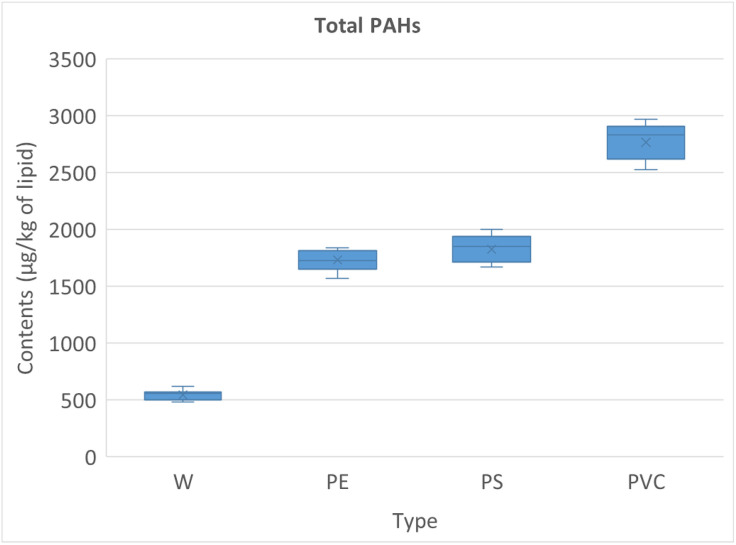
The boxplot of the total PAHs concentrations of the samples. The central line represents the median, the box indicates the interquartile range (IQR), whiskers represent the minimum and maximum values, and “×” denotes the mean.

The boxplot (Fig 4) distributions confirmed these trends, indicating not only elevated mean values but also broader variability in the PVC group compared to the other treatments. These results demonstrate that while wood combustion alone generates substantial amounts of PAHs, the inclusion of plastics in smoking fuels dramatically increases the total burden. In particular, PVC combustion produced the most severe contamination, consistent with its chlorine content and high potential for incomplete combustion by-products.

**Fig 5:**
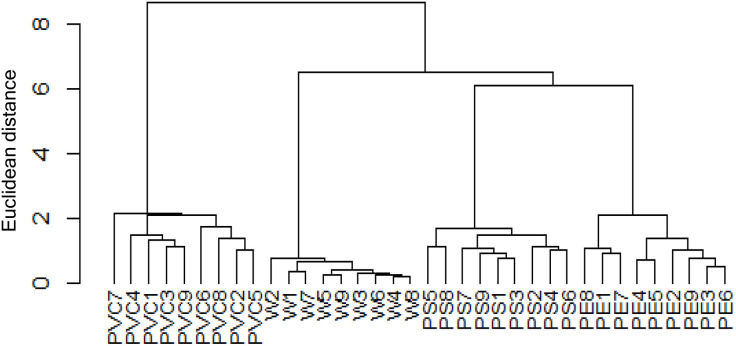
Hierarchical cluster analysis dendrogram of the PAHs in smoked chicken samples, constructed using Euclidean distance and Ward’s method after data standardization.

The hierarchical cluster analysis (HCA) of PAHs further confirmed the separation of samples according to the type of smoking fuel (Fig 5). The dendrogram showed a clear distinction between the PVC group and all other treatments, reflecting its consistently higher PAH burden. The W group clustered tightly and separately, consistent with its comparatively low concentrations. Interestingly, the PE and PS groups formed closely related subclusters, positioned between W and PVC, which is in agreement with their intermediate PAH levels. This clustering pattern mirrors the quantitative results, emphasizing the gradient of contamination (W <<PE ≈ PS < PVC) and highlighting the strong influence of fuel composition on PAH accumulation in smoked chicken meat. When comparing the clustering of PAHs with that of PCBs and PCDDs/Fs, a consistent pattern emerged across all contaminant classes. In both analyses, PVC samples were grouped together and clearly separated from the other treatments, reflecting their unique and more severe contamination profile. Similarly, W samples consistently formed a distinct low-contamination cluster. The PE and PS groups, although distinguishable from each other, were placed in intermediate positions between W and PVC, indicating moderate but elevated contamination compared to clean wood. This parallel clustering pattern across PCBs, PCDDs/Fs, and PAHs underscores the robustness of the results and highlights the strong and consistent effect of fuel composition on the accumulation of multiple classes of hazardous compounds in smoked chicken meat.

To complement the HCA results, principal component analysis (PCA) was applied to explore the contaminant fingerprints associated with different smoking fuels and to identify the compounds most responsible for group separation. PCA is particularly suitable for this dataset because it reduces the dimensionality of multiple correlated PAHs while retaining most of the variance, thereby highlighting the pollutants that differentiate fuel types.

**Fig 6:**
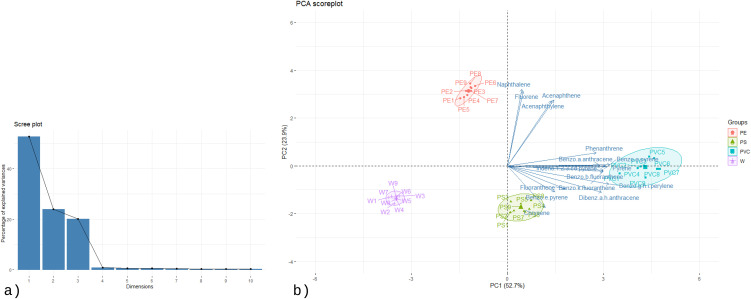
The PCA (a) scree plot and (b) biplot of PAHs concentrations of the samples.

The eigenvalue distribution showed that the first two principal components captured 76.6% of the total variance (PC1: 52.7%, PC2: 23.9%), as illustrated in the scree plot (Fig 6a). The strong dominance of PC1 confirmed that a limited number of pollutants accounted for most of the group differentiation, validating the robustness of the PCA model. The PCA biplot (Fig 6b) revealed distinct clustering of samples according to fuel type and the orientation of compound vectors indicated the characteristic contaminants of each group. The PE group was associated with lighter PAHs such as naphthalene, acenaphthylene, acenaphthene, and fluorene. The PS group was characterized by mid-weight PAHs including fluoranthene, pyrene, benzo[a]anthracene, chrysene, benzo[b]fluoranthene, benzo[k]fluoranthene, and benzo[e]pyrene. In contrast, the PVC group was distinguished by a broader and heavier profile, marked by phenanthrene, fluoranthene, pyrene, benzo[a]anthracene, chrysene, benzo[b]fluoranthene, benzo[k]fluoranthene, benzo[e]pyrene, benzo[a]pyrene, indeno[1,2,3-cd]pyrene, dibenz[a,h]anthracene, and benzo[g,h,i]perylene. Non-parametric Friedman tests were performed to statistically validate these differences among groups. All marker compounds showed highly significant differences (*p* < 0.001) across treatments. These results confirm that the identified compounds are not only characteristic of each fuel type in the PCA biplot but also statistically discriminant between groups.

Taken together, PCA and statistical testing reinforce that fuel composition determines distinct PAH fingerprints in smoked chicken meat: lighter PAHs dominate PE combustion, mid-weight PAHs are associated with PS, and heavier PAHs, particularly highly carcinogenic congeners such as benzo[a]pyrene and indeno[1,2,3-cd]pyrene, are hallmarks of PVC combustion.

## Discussion

The present study demonstrated that the use of plastic-contaminated fuels in traditional smoking markedly altered the contamination profile of smoked chicken meat, resulting in significantly elevated levels of PCBs, PCDD/Fs, and PAHs compared to clean wood. Clean wood generated negligible contamination, whereas the inclusion of plastics, particularly PVC, led to substantial enrichment of toxic congeners. These findings highlight the critical role of fuel composition in determining the chemical safety of smoked foods. Our results showed that smoking with PVC generated the highest levels of both PCDD/Fs and PCBs, followed by PS and PE, while clean wood (W) produced no detectable PCDD/Fs. The clustering patterns observed in both HCA and PCA consistently separated PVC samples due to their broad contaminant spectrum, including twelve PCDD/F congeners and twenty-one PCB congeners. In particular, PCB 189, 101, 180, and 153 emerged as characteristic markers of chlorine-rich combustion. By contrast, PS combustion was distinguished by moderate levels of PCDD/Fs and PCBs, with PCB 66 and 195 significantly enriched. PE combustion was characterized by lighter congeners such as 2,3,7,8-TCDF and 1,2,3,7,8-PeCDF. These results align with earlier findings that waste burning and uncontrolled thermal processes are major sources of PCDD/F and PCB contamination in food products [13,14]. For instance, Hoogenboom et al. (2021) reported unusually high levels of dioxins and PCBs in the meat and fat of free-ranging livestock exposed to contaminated environments, with burning identified as the dominant source [13]. Similarly, studies in Vietnam have shown that dioxin exposure through locally produced food (e.g., free-ranging chicken and duck) poses a major risk to residents in hotspot areas such as Bien Hoa [15]. Our findings suggest that even in non-hotspot contexts, the use of inappropriate fuels such as PVC can generate contamination levels of comparable concern.

In addition to dioxin-related pollutants, PAHs were strongly affected by the type of smoking fuel. Clean wood produced relatively low levels (mean ~544 μg/kg of lipid), whereas PE and PS increased total PAHs more than threefold, and PVC resulted in the highest burden (~2766 μg/kg of lipid), nearly five times greater than W. PCA confirmed that lighter PAHs (naphthalene, acenaphthylene, acenaphthene, fluorene) were characteristic of PE, mid-weight PAHs (fluoranthene, pyrene, chrysene, benzo[e]pyrene) were associated with PS, and heavier carcinogenic PAHs (benzo[a]pyrene, indeno[1,2,3-cd]pyrene, dibenz[a,h]anthracene, benzo[g,h,i]perylene) were dominant in PVC. These results are consistent with reports that smoking conditions and fuel types significantly influence PAH formation in meat and fish products [16]. Duedahl-Olesen et al. (2010) found that hot smoking and the use of certain wood species (e.g., alder) increased PAH levels in smoked fish, while indirect smoking reduced them [16]. Likewise, Cheng et al. (2017) reported detectable levels of carcinogenic PAHs such as benzo[a]pyrene and chrysene in commercial ready-to-eat meats from the UK market [17]. Compared with these studies, the PAH concentrations measured in our PVC-smoked samples were substantially higher, indicating that the addition of plastics to fuel not only changes the PAH profile but also amplifies overall contamination.

The levels of PCBs and PCDD/Fs observed in plastic-smoked samples indicate clear differences in toxicological risk relative to international food safety thresholds. According to Commission Regulation (EU) 2023/915, the maximum level for poultry meat is 1.75 pg WHO-TEQ/g fat for the sum of PCDD/Fs and 3.0 pg WHO-TEQ/g fat [18] for the combined sum of dioxins and dioxin-like PCBs. For comparison with these regulatory limits, the TEQ values calculated from the concentrations presented in S1 Table (reported as ng/kg of lipid, numerically equivalent to pg/g fat) were expressed on a fat basis. In the present study, clean wood (W) samples showed extremely low TEQ values (0.0031–0.0046 pg WHO-TEQ/g fat), while PE-treated samples remained below the regulatory threshold (0.4266–0.5094 pg WHO-TEQ/g fat). In contrast, the PS group exhibited markedly higher TEQ values (1.7417–2.6262 pg WHO-TEQ/g fat), with several samples exceeding the maximum limit and the lowest sample lying close to the threshold. The most severe contamination occurred in the PVC group, where TEQ values ranged from 4.3139 to 4.6666 pg WHO-TEQ/g fat, meaning that all samples exceeded the PCDD/F limit by approximately 2.5-fold and also surpassed the combined PCDD/F + dl-PCB limit of 3.0 pg WHO-TEQ/g fat. These findings provide direct quantitative evidence that combustion of PS and especially PVC can generate smoked poultry products that are non-compliant with EU food safety standards. Comparable concerns have also been reported elsewhere. Ostrich meat and eggs studied in Poland showed elevated dioxin and PCB levels, sometimes exceeding chicken regulatory limits by up to 15-fold [19]. Similarly, a Korean survey of livestock products reported that indicator PCBs such as PCB 28, 52, 101, 138, 153, and 180 were consistently found in chicken fat, with PCB 28 being dominant [20], which parallels our finding of elevated lower-chlorinated congeners in PS-smoked chicken. PAH contamination also raises serious food safety concerns. Our results show that PVC and PS combustion produced precisely these compounds at significantly elevated levels. Previous studies on smoked fish [16] and commercial meats [17] have reported PAH levels ranging from tens to a few hundred μg/kg of lipid, whereas our PVC samples reached nearly 3000 μg/kg of lipid, underscoring the severity of plastic-derived contamination.

Taken together, our results confirm that the inclusion of plastics in smoking fuel leads to a marked increase in persistent organic pollutants (PCBs, PCDD/Fs, and PAHs) in smoked chicken meat, both in terms of total concentrations and specific toxic profiles. These findings are consistent with previous studies linking open burning of contaminated material to elevated levels of such pollutants in meat, eggs, and fish [13–15]. Importantly, the gradient of contamination (W <<PE < PS < PVC) observed across all pollutant groups highlights the direct relationship between fuel composition and food safety.

## Conclusion

This study provides clear evidence that the use of plastic-contaminated fuels in traditional smoking substantially increases the contamination of smoked chicken meat with persistent organic pollutants, including PCBs, PCDD/Fs, and PAHs. Clean wood produced negligible contamination, whereas PE and PS combustion generated distinct sets of marker compounds, and PVC resulted in the most severe and complex pollutant profiles. Both multivariate analyses (HCA, PCA) and statistical tests consistently demonstrated the strong influence of fuel composition on pollutant fingerprints. The contamination gradient observed across all chemical classes (W <<PE < PS < PVC) highlights the urgent need for stricter control of smoking fuels to prevent food contamination and mitigate health risks. Given the carcinogenic nature of several identified congeners (e.g., 2,3,7,8-TCDF, PCB 153, benzo[a]pyrene), the findings underscore that burning plastics during food smoking is a serious food safety hazard with implications for long-term public health.

## Supporting information

S1 Text Experimental. (DOCX)

S1 Fig The PCA loading plot of PCBs and PCDDs/Fs concentrations of the samples. (PPTX)

S2 Fig The PCA loading plot of the PAHs concentrations of the samples. (PPTX)

S1 Table PCBs and PCDDs/Fs concentrations of the samples. (DOCX)

S2 Table Pairwise comparison results (adjusted p-values) for selected PCBs and PCDD/Fs in smoked chicken meat samples across different fuel types (W, PE, PS, PVC). Statistical significance was determined at p < 0.05, indicating that the corresponding PCB or PCDD/F congener exhibited significant differences in concentration between the compared fuel groups. Adjusted p-values were obtained using the Benjamini–Hochberg (BH) method. Values shown in bold indicate statistically significant differences. NA indicates that the p-value could not be computed due to tied or zero differences in paired data.
(DOCX)

S3 Table PAHs concentrations of the samples. (DOCX)

S4 Table Pairwise comparisons were performed using the Wilcoxon signed-rank test with Benjamini–Hochberg correction for polycyclic aromatic hydrocarbons (PAHs) in smoked chicken meat samples across different fuel types (W, PE, PS, PVC). Statistical significance was defined at p < 0.05, indicating that the corresponding PAH congener exhibited significant differences in concentration between the compared fuel groups. Values in bold indicate statistically significant differences.
(DOCX)

## References

[1] Nizio E, Czwartkowski K, Niedbała G. Impact of Smoking Technology on the Quality of Food Products: Absorption of Polycyclic Aromatic Hydrocarbons (PAHs) by Food Products during Smoking. Sustainability. 2023;15(24):16890. doi: 10.3390/su152416890

[2] Tavakoly Sany SB, Hashim R, Salleh A, Rezayi M, Karlen DJ, Razavizadeh BBM, et al. Dioxin risk assessment: mechanisms of action and possible toxicity in human health. Environ Sci Pollut Res Int. 2015;22(24):19434–50. doi: 10.1007/s11356-015-5597-x 26514567

[3] Kirkok SK, Kibet JK, Kinyanjui TK, Okanga FI. A review of persistent organic pollutants: dioxins, furans, and their associated nitrogenated analogues. SN Appl Sci. 2020;2(10). doi: 10.1007/s42452-020-03551-y

[4] Altarawneh M, Dlugogorski BZ, Kennedy EM, Mackie JC. Mechanisms for formation, chlorination, dechlorination and destruction of polychlorinated dibenzo-p-dioxins and dibenzofurans (PCDD/Fs). Progress in Energy and Combustion Science. 2009;35(3):245–74. doi: 10.1016/j.pecs.2008.12.001

[5] IARC Monographs on the Evaluation of Carcinogenic Risks to Humans. WHO; 2012.

[6] Van den Berg M, Birnbaum LS, Denison M, De Vito M, Farland W, Feeley M, et al. The 2005 World Health Organization reevaluation of human and Mammalian toxic equivalency factors for dioxins and dioxin-like compounds. Toxicol Sci. 2006;93(2):223–41. doi: 10.1093/toxsci/kfl055 16829543 PMC2290740

[7] Loganathan BG, Masunaga S. PCBs, dioxins, and furans: human exposure and health effects. In: Gupta RC, editor. Handbook of toxicology of chemical warfare agents. San Diego: Academic Press. 2009:245–53.

[8] Virtanen HE, Koskenniemi JJ, Sundqvist E, Main KM, Kiviranta H, Tuomisto JT, et al. Associations between congenital cryptorchidism in newborn boys and levels of dioxins and PCBs in placenta. Int J Androl. 2012;35(3):283–93. doi: 10.1111/j.1365-2605.2011.01233.x 22150420 PMC3417377

[9] Hennig B, Hammock BD, Slim R, Toborek M, Saraswathi V, Robertson LW. PCB-induced oxidative stress in endothelial cells: modulation by nutrients. Int J Hyg Environ Health. 2002;205(1–2):95–102. doi: 10.1078/1438-4639-00134 12018021

[10] Shertzer HG, Nebert DW, Puga A, Ary M, Sonntag D, Dixon K, et al. Dioxin causes a sustained oxidative stress response in the mouse. Biochem Biophys Res Commun. 1998;253(1):44–8. doi: 10.1006/bbrc.1998.9753 9875217

[11] Uyen NT, Giang DH, Thuy LM, Xuyen NT, Minh NTT, Duong HT, et al. Effect of Combustion Material on the Level of Persistent Organic Pollutants in Smoked Pork. CAC. 2026;22(3):564–76. doi: 10.2174/0115734110357308250119050856

[12] Nguyen Thi X, Nguyen Xuan H, Chu DB, Bui QM, Nguyen TD, Le Hoang TA, et al. Analysis of PCDD/Fs in environmental samples by using gas chromatography in combination with high resolution mass spectrometry: optimization of sample preparation. International Journal of Environmental Analytical Chemistry. 2023;105(2):355–71. doi: 10.1080/03067319.2023.2260987

[13] Hoogenboom RLAP, Dam Gt, van Leeuwen SPJ, van Egmond H, Nicolina J, Dwarkasing AJS. High levels of dioxins and PCBs in meat, fat and livers of free ranging pigs, goats, sheep and cows from the island of Curaçao. Chemosphere. 2021;263:art.ID. 128057. doi: 10.1016/j.chemosphere.2020.128057 33297065

[14] Fernández-González R, Yebra-Pimentel I, Martínez-Carballo E, Simal-Gándara J. A Critical Review about Human Exposure to Polychlorinated Dibenzo-p-Dioxins (PCDDs), Polychlorinated Dibenzofurans (PCDFs) and Polychlorinated Biphenyls (PCBs) through Foods. Crit Rev Food Sci Nutr. 2015;55(11):1590–617. doi: 10.1080/10408398.2012.710279 24279584

[15] Tuyet-Hanh TT, Vu-Anh L, Ngoc-Bich N, Tenkate T. Environmental health risk assessment of dioxin exposure through foods in a dioxin hot spot-Bien Hoa City, Vietnam. Int J Environ Res Public Health. 2010;7(5):2395–406. doi: 10.3390/ijerph7052395 20623031 PMC2898056

[16] Duedahl-Olesen L, Christensen JH, Højgård A, Granby K, Timm-Heinrich M. Influence of smoking parameters on the concentration of polycyclic aromatic hydrocarbons (PAHs) in Danish smoked fish. Food Addit Contam Part A Chem Anal Control Expo Risk Assess. 2010;27(9):1294–305. doi: 10.1080/19440049.2010.487074 20640961

[17] Lu F, Kuhnle GK, Cheng Q. Heterocyclic amines and polycyclic aromatic hydrocarbons in commercial ready-to-eat meat products on UK market. Food Control. 2017;73:306–15. doi: 10.1016/j.foodcont.2016.08.021

[18] European Commission. Commission Regulation (EU) 2023/915 of 25 April 2023 on maximum levels for certain contaminants in food and repealing Regulation (EC) No 1881/2006. Official Journal of the European Union. 2023:55–66.

[19] Piskorska-Pliszczynska J, Strucinski P, Mikolajczyk S, Pajurek M, Maszewski S, Pietron W. Dioxins and PCBs in ostrich meat and eggs: levels and implications. Food Addit Contam Part A Chem Anal Control Expo Risk Assess. 2017;34(12):2190–200. doi: 10.1080/19440049.2017.1364871 28795866

[20] Kim M, Kim S, Yun S, Lee M, Cho B, Park J, et al. Comparison of seven indicator PCBs and three coplanar PCBs in beef, pork, and chicken fat. Chemosphere. 2004;54(10):1533–8. doi: 10.1016/j.chemosphere.2003.10.044 14659955

