# Supplementary material for: Effect of plastic composition in the combustion material on the Persistent Organic Pollutant content in smoked chicken meat
Source: PLoS One. 2026 Jun 3;21(6):e0350345. doi: 10.1371/journal.pone.0350345 (PMC13232828; doi:10.1371/journal.pone.0350345)
Supplement: S1 Text — (DOCX) [file pone.0350345.s001.docx]

**EXPERIMENTAL**

## Reagents and chemicals

### For PAH and PCB analysis

Native standard mixtures were used for external calibration in the analysis of polycyclic aromatic hydrocarbons (PAHs) and polychlorinated biphenyls (PCBs). For PAHs, the PTM PAH Mix containing 17 target compounds at a concentration of 2000 µg/mL was obtained from Sigma-Aldrich (Germany). For PCBs, a WHO/NIST/NOAA mixture comprising 28 congeners at 10 µg/mL in *n*-hexane was purchased from AccuStandard (USA).

*List of the PAHs standards*

| **No.** | **Name of compound** | **No.** | **Name of compound** |
| --- | --- | --- | --- |
|  | Naphthalene |  | Benzo[b]fluoranthene |
|  | Acenaphthylene |  | Benzo[k]fluoranthene |
|  | Acenaphthene |  | Benzo[e]pyrene |
|  | Fluorene |  | Benzo[a]pyrene |
|  | Phenanthrene |  | Indeno[1,2,3-cd]pyrene |
|  | Anthracene |  | Dibenz[a,h]anthracene |
|  | Fluoranthene |  | Benzo[g,h,i]perylene |
|  | Pyrene |  |  |
|  | Benzo[a]anthracene |  |  |
|  | Chrysene |  |  |

Isotopically labelled internal standards (IS) were applied throughout the analytical procedure to correct for recovery efficiency and matrix effects. For PAHs, the EPA 8270 IS mixture, including naphthalene-*d*_8_, phenanthrene-d₁₀, chrysene-*d*_12_, and related compounds, was used. For PCBs, a mixture of seven fully ^13^C-labelled congeners (PCB-28, PCB-52, PCB-101, PCB-138, PCB-153, PCB-180, and PCB-209) was employed as internal standards during sample preparation. In addition, a separate ^13^C-labelled mixture containing PCB-77, PCB-81, PCB-123, PCB-126, and PCB-169 was used exclusively as injection internal standards.

Working standard solutions were freshly prepared on a weekly basis in *n*-hexane at concentrations ranging from 2–2000 ng/mL for PAHs and 0.2–200 ng/mL for PCBs, and were used solely for external calibration. All solvents employed for PAH and PCB analysis, including *n*-hexane, acetone, dichloromethane, and methanol, as well as other reagents such as anhydrous sodium sulfate (Na_2_SO_4_), silica gel, and concentrated sulfuric acid (98%), were of analytical grade and high purity, and were supplied by Merck (Singapore). Anhydrous Na_2_SO_4_ was baked at 450 °C for 3 h to remove organic contaminants and subsequently stored in an amber glass bottle in a desiccator until use.

### For PCDD/F analysis

Quantification and recovery correction of polychlorinated dibenzo-p-dioxins and dibenzofurans (PCDD/Fs) were carried out using the isotope-dilution method in accordance with US EPA Method 1613B and Method 23. The standard sets consisted of native calibration solutions for the 17 toxic PCDD/F congeners, fully ^13^C-labelled internal standards spiked prior to extraction to monitor overall method recovery, and ^13^C-labelled syringe standards added after sample clean-up to assess injection variability.

Nitrogen and helium gases with a purity of 99.99% were used during the analytical process. Solvents for PCDD/F analysis, including acetone, dichloromethane, toluene, nonane, and n-hexane, were of dioxin analysis grade. Chemical reagents such as anhydrous sodium sulfate, potassium hydroxide, sulfuric acid, and silver nitrate were of chromatography grade. All solvents and chemicals were obtained from Merck (Germany). Silica gel (60–200 mesh) for open-column chromatography was purchased from VWR International (USA), while activated carbon impregnated on silica gel (P/N 019-11941) was supplied by Wako (Japan).

## Sample pretreatments and chromatographic conditions

The isotopic dilution gas chromatography-tandem high-resolution mass spectrometry was employed to quantify the concentrations of PAHs, PCBs, and PCDD/Fs in smoked chicken samples. However, sample pretreatment processes and the analytical instrumentation were adjusted according to the target compounds. Detailed information for each type of analyzed POPs is provided below.

### For PAH and PCB analysis

#### Sample extraction

An accurately weighed 5 g portion of each freeze-dried smoked chicken sample was transferred into an extraction cell and fortified with 20 µL of a ^13^C-labelled internal standard mixture (10 pg µL⁻¹) containing the target PAH and PCB congeners. Sample extraction was performed using an accelerated solvent extraction system (E-916, Büchi, Switzerland). A binary solvent mixture of toluene and acetone (7:3, v/v) was employed as the extraction medium. The extraction process was conducted at an elevated temperature of 120 °C and a pressure of 100 bar, with a static extraction period of 15 min per cycle. A total of three static extraction cycles were applied to ensure exhaustive recovery of the analytes from the matrix.

Following extraction, the combined extract was quantitatively divided into two fractions. One aliquot was reserved for lipid content determination, while the remaining portion was subjected to further clean-up and instrumental analysis for the determination of persistent organic pollutants (POPs), including PAHs and PCBs.

#### Determination of fat content

Total lipid content was determined gravimetrically. The designated extract fraction was evaporated to dryness under reduced pressure to remove the extraction solvent completely. The residue was then dried in a temperature-controlled oven at 80 °C for 5 h to eliminate residual moisture and volatile components. After cooling to room temperature in a desiccator, the mass of the remaining residue was recorded and used to calculate the lipid content of each sample.

#### Purification and enrichment

The extract allocated for POP analysis was concentrated to approximately 0.5 mL under a gentle stream of high-purity nitrogen, followed by solvent exchange to *n*-hexane. Sample clean-up was carried out using an acidified silica gel solid-phase extraction (SPE) cartridge to remove co-extracted lipids and matrix interferences. Prior to sample loading, the SPE cartridge was sequentially conditioned with 10 mL of methanol and 10 mL of *n*-hexane at a flow rate of approximately 3 mL min⁻¹. The sample extract was then applied to the cartridge and eluted with 12 mL of a hexane/dichloromethane mixture (1:1, v/v). The collected eluate was subsequently concentrated to near dryness under nitrogen and reconstituted to a final volume of exactly 1.0 mL with *n*-hexane.

#### GC-MS analysis and instrumentation

Quantitative determination of PAHs and PCBs was performed using gas chromatography coupled with electron impact tandem mass spectrometry (GC–EI–MS/MS). The analytical system consisted of a Trace 1310 gas chromatograph (Thermo Scientific, USA) equipped with a TriPlus RSH liquid autosampler and interfaced with a TSQ 9000 triple quadrupole mass spectrometer (Thermo Scientific, USA). Instrumental performance and sensitivity were routinely verified using FC-43 as a tuning compound. Chromatographic separation of the 17 PAHs and 28 PCB congeners was achieved on a DB-5MS capillary column (30 m × 0.25 mm i.d., 0.25 µm film thickness; 5% methylphenyl polysiloxane stationary phase). A sample volume of 1 µL was introduced into the GC system in splitless injection mode, with the injector temperature maintained at 280 °C. Helium (99.999% purity) was used as the carrier gas at a constant flow rate of 1.0 mL min⁻¹ throughout the chromatographic run. The oven temperature program was initiated at 100 °C and held for 3 min, followed by a linear increase to 210 °C at 15 °C min⁻¹. The temperature was then ramped to 300 °C at a rate of 10 °C min⁻¹ and held for 15 min to ensure complete elution of high-boiling compounds and conditioning of the column. The GC–MS interface temperature was set at 300 °C, while the electron ionization (EI) source and quadrupole temperatures were maintained at 280 °C and 150 °C, respectively. Electron impact ionization was operated at 70 eV. Detection of PAHs and PCBs was conducted in positive EI mode using selected reaction monitoring (SRM), providing high sensitivity and selectivity. Quantification of target analytes in real samples was achieved using isotope-dilution calibration curves based on the corresponding ^13^C-labelled internal standards.

- - 1. *Linearity, method detection limit (MDL), method quantitation limit (MQL), and recovery performance for PAHs and PCBs*

Analytical performance parameters, including linearity, method sensitivity, and recovery efficiency for PAHs and PCBs, were systematically assessed in accordance with relevant US EPA validation guidelines. Quantitative calibration was achieved using multi-point calibration curves constructed from native reference standards in combination with corresponding ^13^C-labelled internal standards. Across the entire calibration range, all target analytes exhibited highly linear responses, with coefficients of determination (R²) consistently exceeding 0.999, indicating excellent instrumental linearity and robustness of the calibration model.

Method detection limits (MDLs) and method quantification limits (MQLs) were estimated based on the standard deviation of replicate low-level measurements (σ) and the slope of the calibration curve (S), following the EPA-recommended equations (MDL = 3.3σ/S; MQL = 10σ/S). To ensure consistency with the reporting format adopted throughout the manuscript, all sensitivity parameters are expressed on a lipid-weight basis (ppb lipid). For PAHs, MDL values were found to range from 1.1 to 2.0 ppb lipid, while the corresponding MQLs ranged from 3.7 to 6.7 ppb lipid. In the case of PCBs, substantially lower detection limits were achieved, with MDLs generally between 0.001 and 0.003 ppb lipid and MQLs between 0.003 and 0.010 ppb lipid, demonstrating the suitability of the method for ultra-trace analysis of PCBs in smoked meat matrices.

Method accuracy and extraction efficiency were further evaluated through recovery experiments using blank meat samples fortified with isotopically labelled internal standards (n = 5). The recoveries of ^13^C-labelled PAH internal standards ranged from 74% to 111%, fully complying with the acceptance criteria specified in EPA Method 8270 (70–120%). Similarly, recoveries of ^13^C-labelled PCB internal standards varied between 63% and 110%, which fall within the quality control limits (60–120%) required for isotope-dilution PCB analysis. Overall, these recovery results confirm the effectiveness of the extraction and clean-up procedures and demonstrate the reliability and accuracy of the analytical method for simultaneous determination of PAHs and PCBs in complex, lipid-rich food matrices.

### For PCDD/F analysis

#### Sample extraction

Extraction for PCDD/F analysis was followed by a protocol analogous to the extraction process designated for PAH and PCB congeners. However, before loading the sample in the E-916 accelerated solvent extractor, isotopically labeled PCDD/Fs standard solution was spiked into the freeze-dried samples at a volume of 50 µL (2 pg/µL).

The spiked ^13^C-labeled isotope PCDD/F standards were used before extraction to evaluate the extraction efficiency of the method. This procedure was carried out in accordance with Method 1613B, as stipulated by the United States Environmental Protection Agency (US EPA) in 1991.

#### Fat removal

The remaining part of the sample extract should be removed fat before purification and enrichment. It was transferred to a centrifuge tube along with n-hexane. After dropping concentrated sulfuric acid slowly, the centrifuge tube was centrifuged for 5 minutes. The upper n-hexane layer was transferred to another test tube and then washed with deionised water in triplicate to remove all sulfuric acid from the analyzed samples.

#### Purification and enrichment

The sample was placed into a multilayer column coupled with the activated carbon column to purify the sample. After that, the clean extract was continuously evaporated under lower pressure to a volume of approximately 1mL and evaporated in nitrogen gas. Then, it was mixed with 5 µL of the IS at a concentration of 2 pg/µL and made up to the volume of 10 µL with nonane. All sample was filtered through a 0.22 µm membrane before analysis in the GC/MS system.

#### GC-MS analysis and instrument

The analysis process for PCDD/Fs congeners was performed on a gas chromatographic system similar to the equipment used for the quantification of PAHs and PCBs. However, instead of electron impact tandem mass spectrometry, it was equipped with a high-resolution mass spectrometer (Magnetic Sector/Double-Focusing System, Thermo Scientific, USA). In addition, the separation of 17 PCDD/F congeners was achieved on the TG-Dioxin (60m $\times$ 0,25 mm I.D. $\times$ 0.25 µm, P/N: 122-5532) column.

They were separated by programmed temperature as follows: the temperature was kept at 140°C for 2 min, heated from 14°C to 228°C at 40°C/min and from 228°C to 290°C at 1.6°C/min, heated from 290°C to 315°C at 5°C/min, then kept at constant at 315°C for 9 min and then cooled down to the beginning of the temperature program. The solvent delay was set to 17 min. The HR/MS was regularly checked by using the tuning solution (FC-43) in terms of mass accuracy and mass resolution efficiency. The resolution of mass spectrometry was set at 10,000 at 10% of peak height. The following conditions were applied for dioxin separation and detection: selective ion monitoring mode, helium as the carrier gas at a constant flow (1.2 mL/min), splitless injection at 250°C, interface temperature at 260°C, and transfer line and ion source temperatures were 280°C and 250°C, respectively.

The SRM mode was also used to detect and quantify PCDD/Fs in this study. They were identified by relative retention times and based on two characteristic masses and the ion ratio of two ions' association with measurement uncertainty. Quantitative analysis was performed by the standard internal method, following the US EPA method 1613B and the US EPA method 23.

- - 1. *Linearity, method detection limit (MDL), method quantitation limit (MQL), and recovery performance for PCDD/Fs*.

The linearity and detection performance of the analytical method for polychlorinated dibenzo-*p*-dioxins and dibenzofurans (PCDD/Fs) were systematically evaluated in compliance with the requirements of US EPA Methods 1613B and 23. Quantification was based on multi-point calibration curves constructed using both native congeners and their corresponding ^13^C-labelled analogues. All calibration models exhibited excellent response stability over the evaluated concentration ranges, with coefficients of determination (R²) consistently between 0.99982 and 0.99999, indicating highly reliable instrumental linearity.

Method detection limits (MDLs) and method quantification limits (MQLs) were calculated according to EPA-recommended statistical approaches, using the standard deviation of replicate low-level measurements (σ) and the slope of the calibration curve (S), following the equations MDL = 3.3σ/S and MQL = 10σ/S. To ensure consistency with the reporting framework adopted in this study, all sensitivity parameters are expressed on a lipid-normalized basis (ppt lipid). For the 17 toxic PCDD/F congeners, MDL values ranged from 0.10 to 0.33 ppt lipid, while corresponding MQLs varied between 0.35 and 1.06 ppt lipid, demonstrating sufficient analytical sensitivity for high-resolution determination of dioxins and furans in complex food matrices.

Method accuracy and extraction efficiency were further assessed through recovery experiments using ^13^C-labelled internal standards added prior to the extraction step. The mean recoveries obtained for individual congeners ranged from 69% to 108%, which fall well within the acceptance criteria of 60–120% specified by the EPA for isotope-dilution-based PCDD/F analysis. Collectively, these validation results confirm the effectiveness of the extraction and clean-up procedures and demonstrate the robustness and reliability of the isotope-dilution quantification approach for PCDD/F determination in smoked chicken samples.

## Data processing analysis

Data processing and statistical analyses are described in detail in the main Methods section.
