# Supplementary figures and images for: Effect of plastic composition in the combustion material on the Persistent Organic Pollutant content in smoked chicken meat
Source: PLoS One. 2026 Jun 3;21(6):e0350345. doi: 10.1371/journal.pone.0350345 (PMC13232828; doi:10.1371/journal.pone.0350345)

## Slide 1
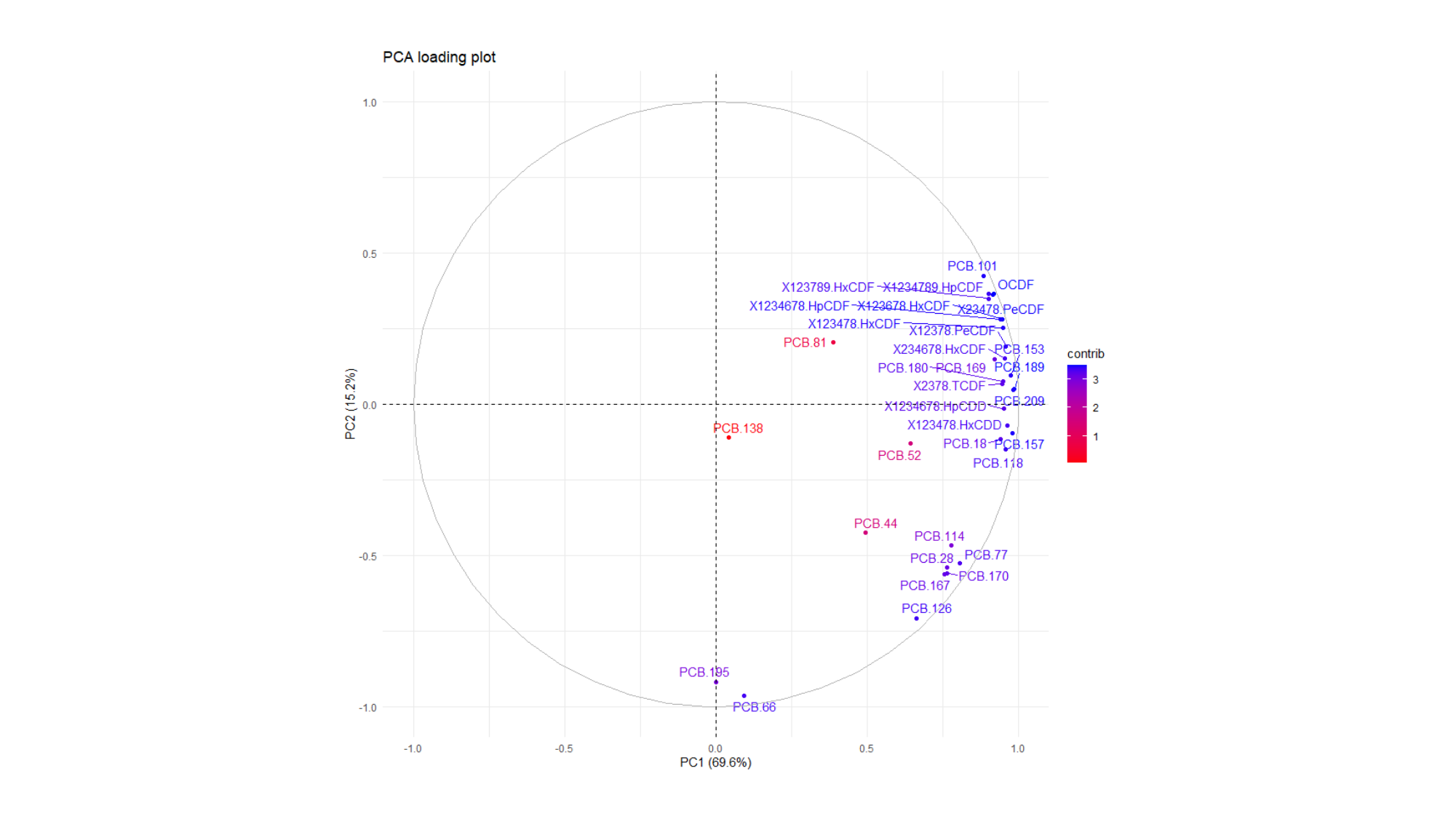

Supplement: S1 Fig — (PPTX) [file pone.0350345.s002.pptx]

## Slide 1
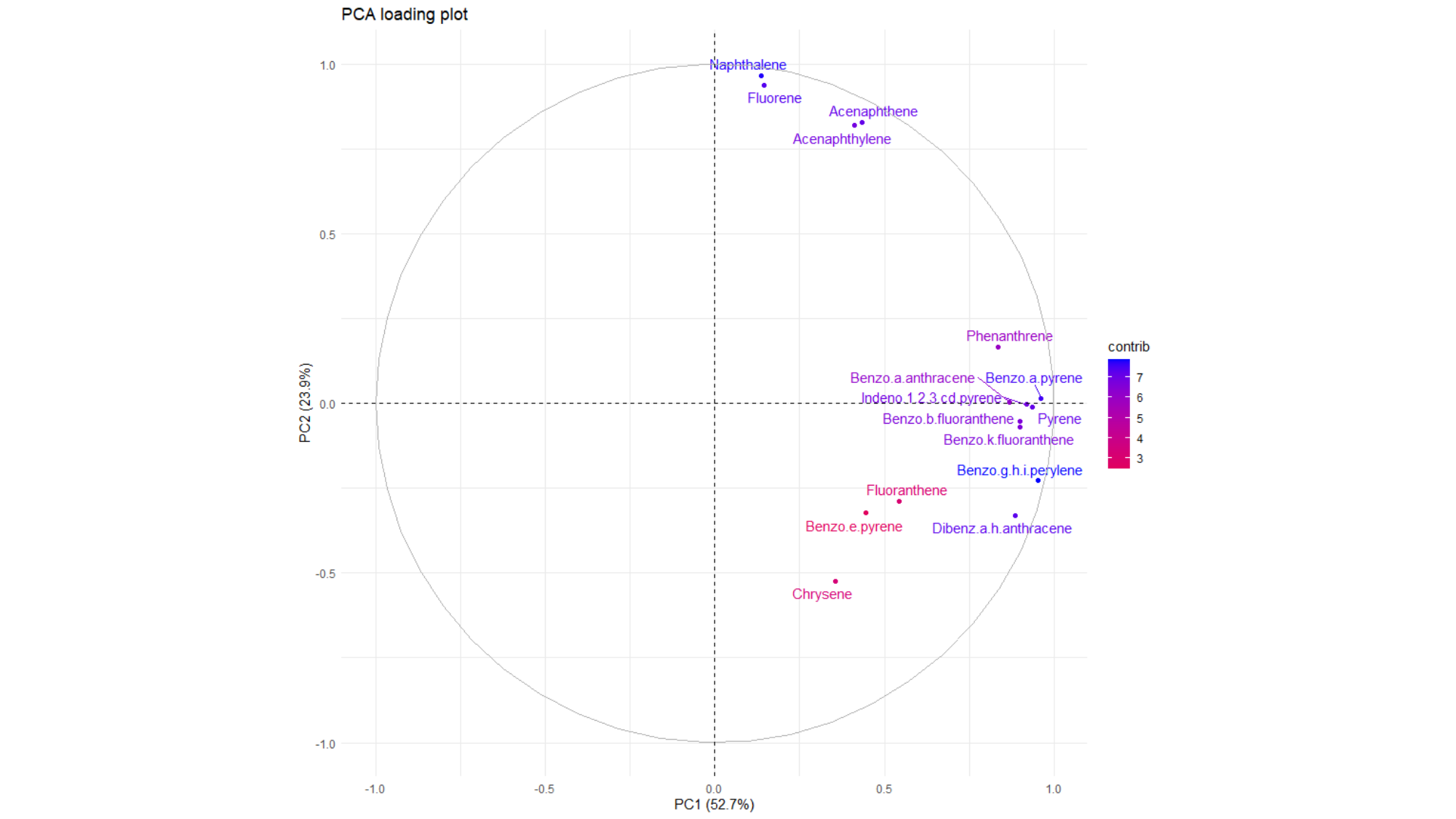

Supplement: S2 Fig — (PPTX) [file pone.0350345.s003.pptx]
