# Supplementary material for: Effect of plastic composition in the combustion material on the Persistent Organic Pollutant content in smoked chicken meat
Source: PLoS One. 2026 Jun 3;21(6):e0350345. doi: 10.1371/journal.pone.0350345 (PMC13232828; doi:10.1371/journal.pone.0350345)
Supplement: S1 Table — (DOCX) [file pone.0350345.s004.docx]

**Table S1. PCBs and PCDDs/Fs concentrations of the samples**

|  | **Concentration (Mean ± SD, ng/kg of lipid)** | | | | | | |
| --- | --- | --- | --- | --- | --- | --- | --- |
| **Sample** | **PCB 18** | **PCB 28** | **PCB 44** | **PCB 52** | **PCB 66** | **PCB 77** | **PCB 81** |
| **W1** | 11.57 ± 1.50 | 12.32 ± 1.73 | 11.78 ± 1.77 | 11.11 ± 1.69 | N.D. | 9.89 ± 1.51 | N.D. |
| **W2** | 15.01 ± 2.06 | 11.04 ± 1.32 | 13.01 ± 2.12 | 11.64 ± 1.87 | N.D. | 7.72 ± 0.86 | N.D. |
| **W3** | 13.93 ± 2.19 | 15.68 ± 1.81 | 14.91 ± 1.55 | 10.04 ± 1.72 | N.D. | 10.23 ± 1.37 | N.D. |
| **W4** | 14.43 ± 1.60 | 15.29 ± 2.23 | 11.70 ± 1.65 | 13.04 ± 1.30 | N.D. | 12.87 ± 1.36 | N.D. |
| **W5** | 12.31 ± 1.63 | 16.29 ± 1.86 | 14.94 ± 2.37 | 13.58 ± 2.12 | N.D. | 10.17 ± 1.53 | N.D. |
| **W6** | 15.35 ± 1.63 | 14.34 ± 2.07 | 11.23 ± 1.76 | 13.98 ± 2.34 | N.D. | 12.08 ± 1.98 | N.D. |
| **W7** | 9.64 ± 0.88 | 14.73 ± 1.94 | 9.95 ± 1.16 | 9.47 ± 0.95 | N.D. | 9.51 ± 0.93 | N.D. |
| **W8** | 14.58 ± 1.86 | 12.20 ± 1.24 | 11.71 ± 1.74 | 8.71 ± 1.31 | N.D. | 10.85 ± 1.21 | N.D. |
| **W9** | 11.83 ± 1.18 | 13.75 ± 1.69 | 15.13 ± 1.62 | 11.39 ± 0.97 | N.D. | 9.74 ± 0.81 | N.D. |
| **PE1** | 23.67 ± 2.41 | 76.06 ± 5.88 | 34.97 ± 4.04 | 45.93 ± 3.85 | N.D. | 15.90 ± 1.70 | 11.78 ± 1.85 |
| **PE2** | 23.03 ± 2.50 | 74.33 ± 9.74 | 34.25 ± 5.65 | 43.91 ± 5.58 | N.D. | 17.79 ± 1.47 | 10.99 ± 1.03 |
| **PE3** | 17.01 ± 2.25 | 90.70 ± 11.60 | 37.45 ± 5.64 | 53.44 ± 5.43 | N.D. | 11.52 ± 1.72 | 10.05 ± 0.86 |
| **PE4** | 26.68 ± 3.44 | 59.54 ± 9.79 | 42.04 ± 7.08 | 57.27 ± 4.56 | N.D. | 20.86 ± 2.25 | 13.59 ± 2.06 |
| **PE5** | 23.42 ± 2.78 | 87.42 ± 8.98 | 23.87 ± 3.45 | 34.29 ± 5.55 | N.D. | 14.89 ± 2.40 | 15.46 ± 1.45 |
| **PE6** | 19.33 ± 1.73 | 76.22 ± 10.12 | 27.86 ± 2.92 | 41.11 ± 6.65 | N.D. | 12.41 ± 1.81 | 13.97 ± 1.61 |
| **PE7** | 19.04 ± 1.74 | 92.75 ± 8.43 | 40.47 ± 4.55 | 38.17 ± 4.37 | N.D. | 15.72 ± 1.24 | 13.21 ± 1.08 |
| **PE8** | 27.99 ± 2.53 | 100.33 ± 12.89 | 38.04 ± 3.93 | 34.76 ± 3.00 | N.D. | 11.54 ± 1.79 | 10.31 ± 1.71 |
| **PE9** | 16.87 ± 2.64 | 76.64 ± 12.48 | 43.99 ± 4.48 | 55.28 ± 5.52 | N.D. | 19.16 ± 2.00 | 12.70 ± 2.00 |
| **PS1** | 32.60 ± 2.49 | 88.42 ± 13.67 | 35.30 ± 4.26 | 33.03 ± 3.68 | 105.00 ± 17.27 | 93.70 ± 14.17 | 2.08 ± 0.17 |
| **PS2** | 33.92 ± 2.80 | 170.09 ± 28.85 | 33.32 ± 2.96 | 32.60 ± 4.77 | 115.70 ± 15.43 | 76.85 ± 6.95 | 3.10 ± 0.51 |
| **PS3** | 43.14 ± 3.72 | 221.23 ± 31.87 | 32.43 ± 3.96 | 39.08 ± 5.67 | 102.88 ± 15.01 | 88.41 ± 11.74 | 3.60 ± 0.60 |
| **PS4** | 40.87 ± 6.76 | 146.13 ± 22.00 | 28.19 ± 3.83 | 36.17 ± 2.75 | 139.12 ± 11.29 | 85.52 ± 10.82 | 2.49 ± 0.29 |
| **PS5** | 30.54 ± 4.46 | 184.66 ± 16.92 | 41.30 ± 5.54 | 29.87 ± 3.52 | 114.89 ± 14.94 | 97.30 ± 15.56 | 3.85 ± 0.47 |
| **PS6** | 34.51 ± 4.99 | 145.69 ± 12.55 | 36.50 ± 5.48 | 35.57 ± 5.52 | 107.42 ± 16.98 | 77.17 ± 7.19 | 2.95 ± 0.30 |
| **PS7** | 27.17 ± 4.54 | 218.18 ± 16.95 | 41.11 ± 3.84 | 29.12 ± 2.35 | 96.14 ± 14.33 | 70.50 ± 8.35 | 2.42 ± 0.20 |
| **PS8** | 37.80 ± 3.18 | 200.74 ± 29.45 | 26.79 ± 3.24 | 32.87 ± 4.84 | 131.48 ± 10.22 | 92.17 ± 13.13 | 3.33 ± 0.55 |
| **PS9** | 28.44 ± 3.79 | 192.93 ± 17.14 | 29.09 ± 4.82 | 38.08 ± 4.41 | 113.29 ± 17.24 | 85.27 ± 7.62 | 2.57 ± 0.41 |
| **PVC1** | 56.86 ± 6.09 | 181.95 ± 22.99 | 33.75 ± 4.05 | 44.12 ± 4.68 | 10.89 ± 1.06 | 86.28 ± 13.01 | 8.80 ± 1.08 |
| **PVC2** | 35.44 ± 5.38 | 104.82 ± 12.48 | 36.29 ± 2.88 | 56.09 ± 5.94 | 12.18 ± 1.37 | 72.34 ± 10.80 | 6.10 ± 0.96 |
| **PVC3** | 50.13 ± 7.86 | 168.93 ± 19.83 | 31.66 ± 4.51 | 47.19 ± 5.58 | 12.09 ± 1.50 | 96.14 ± 14.05 | 9.18 ± 1.36 |
| **PVC4** | 63.89 ± 5.77 | 194.11 ± 15.41 | 30.24 ± 3.75 | 41.71 ± 4.37 | 10.62 ± 0.92 | 81.12 ± 12.67 | 9.54 ± 1.15 |
| **PVC5** | 52.91 ± 5.22 | 161.22 ± 20.27 | 30.49 ± 2.54 | 44.94 ± 6.55 | 10.22 ± 1.52 | 81.96 ± 12.44 | 8.73 ± 1.03 |
| **PVC6** | 59.47 ± 5.44 | 176.00 ± 14.35 | 36.24 ± 5.00 | 48.57 ± 5.61 | 9.95 ± 1.36 | 89.93 ± 9.44 | 9.10 ± 0.91 |
| **PVC7** | 51.09 ± 5.16 | 189.83 ± 32.46 | 31.57 ± 2.59 | 47.66 ± 7.86 | 12.23 ± 1.72 | 83.98 ± 11.78 | 8.94 ± 1.14 |
| **PVC8** | 53.63 ± 8.56 | 190.35 ± 18.64 | 30.78 ± 4.59 | 40.56 ± 6.59 | 9.66 ± 1.38 | 87.01 ± 11.86 | 7.68 ± 0.88 |
| **PVC9** | 50.12 ± 4.50 | 166.93 ± 14.00 | 32.34 ± 3.51 | 39.02 ± 4.12 | 11.59 ± 1.38 | 93.91 ± 15.05 | 9.61 ± 1.12 |
|  | **Concentration (Mean ± SD, ng/kg of lipid)** | | | | | | |
| **Sample** | **PCB 101** | **PCB 118** | **PCB 114** | **PCB 126** | **PCB 138** | **PCB 153** | **PCB 157** |
| **W1** | 10.80 ± 1.44 | N.D. | 11.04 ± 1.45 | N.D. | 3.43 ± 0.48 | N.D. | N.D. |
| **W2** | 12.17 ± 1.86 | N.D. | 13.58 ± 1.55 | N.D. | 11.70 ± 1.84 | N.D. | N.D. |
| **W3** | 14.00 ± 1.79 | N.D. | 12.96 ± 2.09 | N.D. | 4.30 ± 0.52 | N.D. | N.D. |
| **W4** | 9.98 ± 1.52 | N.D. | 12.50 ± 1.12 | N.D. | 4.15 ± 0.56 | N.D. | N.D. |
| **W5** | 8.50 ± 1.09 | N.D. | 13.90 ± 1.15 | N.D. | 4.29 ± 0.54 | N.D. | N.D. |
| **W6** | 12.40 ± 1.33 | N.D. | 14.16 ± 1.24 | N.D. | 4.24 ± 0.66 | N.D. | N.D. |
| **W7** | 9.95 ± 1.63 | N.D. | 14.03 ± 1.38 | N.D. | 2.72 ± 0.37 | N.D. | N.D. |
| **W8** | 10.23 ± 1.74 | N.D. | 14.13 ± 1.84 | N.D. | 3.35 ± 0.57 | N.D. | N.D. |
| **W9** | 9.73 ± 0.90 | N.D. | 9.35 ± 0.81 | N.D. | 3.88 ± 0.59 | N.D. | N.D. |
| **PE1** | 33.42 ± 4.24 | 21.52 ± 2.14 | 23.14 ± 3.66 | 4.42 ± 0.39 | 23.07 ± 3.75 | 8.15 ± 0.86 | 4.50 ± 0.58 |
| **PE2** | 30.65 ± 4.31 | 19.20 ± 2.13 | 22.87 ± 2.22 | 4.38 ± 0.37 | 24.61 ± 4.08 | 8.22 ± 0.87 | 4.32 ± 0.33 |
| **PE3** | 27.78 ± 4.54 | 15.29 ± 2.47 | 18.21 ± 3.01 | 4.59 ± 0.55 | 22.97 ± 3.68 | 7.33 ± 1.00 | 3.56 ± 0.38 |
| **PE4** | 29.77 ± 4.00 | 15.57 ± 1.88 | 21.22 ± 3.41 | 5.02 ± 0.42 | 21.01 ± 1.67 | 6.28 ± 0.81 | 5.78 ± 0.67 |
| **PE5** | 24.09 ± 2.52 | 20.64 ± 1.87 | 25.83 ± 3.39 | 5.49 ± 0.70 | 18.42 ± 2.83 | 8.94 ± 0.77 | 5.41 ± 0.60 |
| **PE6** | 43.12 ± 5.80 | 19.97 ± 1.70 | 28.97 ± 4.90 | 4.58 ± 0.36 | 25.67 ± 2.77 | 9.34 ± 0.80 | 5.72 ± 0.46 |
| **PE7** | 31.25 ± 4.92 | 26.54 ± 3.23 | 19.04 ± 1.90 | 5.09 ± 0.59 | 20.06 ± 1.69 | 7.11 ± 1.15 | 4.88 ± 0.77 |
| **PE8** | 38.19 ± 5.44 | 18.76 ± 2.83 | 25.99 ± 3.89 | 5.84 ± 0.52 | 19.19 ± 1.74 | 6.71 ± 1.10 | 3.64 ± 0.61 |
| **PE9** | 38.85 ± 6.50 | 25.46 ± 2.65 | 17.42 ± 1.55 | 4.33 ± 0.50 | 20.66 ± 1.74 | 8.62 ± 0.74 | 6.00 ± 0.53 |
| **PS1** | 23.56 ± 3.53 | 24.96 ± 4.05 | 31.74 ± 3.03 | 23.29 ± 2.44 | 4.80 ± 0.70 | 11.84 ± 1.25 | 8.27 ± 1.08 |
| **PS2** | 10.75 ± 1.22 | 28.80 ± 3.26 | 28.97 ± 3.28 | 32.38 ± 3.57 | 11.95 ± 0.99 | 7.89 ± 1.25 | 8.41 ± 0.86 |
| **PS3** | 9.01 ± 0.95 | 27.45 ± 3.01 | 26.13 ± 3.10 | 26.53 ± 2.96 | 12.06 ± 1.39 | 9.86 ± 1.30 | 7.48 ± 1.09 |
| **PS4** | 11.53 ± 1.96 | 32.68 ± 4.63 | 33.86 ± 4.64 | 27.28 ± 3.25 | 9.81 ± 0.92 | 6.19 ± 0.97 | 10.89 ± 1.26 |
| **PS5** | 8.30 ± 0.92 | 24.91 ± 2.38 | 37.78 ± 5.58 | 41.12 ± 5.06 | 14.53 ± 2.06 | 9.35 ± 0.88 | 9.13 ± 1.41 |
| **PS6** | 10.34 ± 1.01 | 29.43 ± 3.00 | 37.85 ± 5.09 | 36.33 ± 4.91 | 11.42 ± 0.93 | 6.64 ± 0.55 | 10.90 ± 1.72 |
| **PS7** | 11.75 ± 1.72 | 31.36 ± 3.43 | 31.54 ± 2.52 | 33.32 ± 3.24 | 15.19 ± 2.01 | 6.08 ± 0.72 | 10.37 ± 0.80 |
| **PS8** | 9.53 ± 1.22 | 36.23 ± 5.00 | 38.09 ± 5.25 | 42.60 ± 6.68 | 9.69 ± 1.29 | 7.21 ± 0.64 | 6.82 ± 0.57 |
| **PS9** | 10.21 ± 0.94 | 23.91 ± 3.29 | 30.16 ± 3.70 | 38.51 ± 4.36 | 11.74 ± 1.93 | 6.79 ± 0.94 | 10.53 ± 0.88 |
| **PVC1** | 92.94 ± 7.15 | 50.95 ± 4.63 | 33.50 ± 2.63 | 25.65 ± 2.32 | 10.93 ± 1.16 | 23.29 ± 2.42 | 18.24 ± 2.10 |
| **PVC2** | 83.05 ± 8.21 | 61.25 ± 4.80 | 51.89 ± 5.33 | 33.46 ± 3.19 | 11.76 ± 1.95 | 21.19 ± 3.61 | 22.24 ± 1.76 |
| **PVC3** | 84.66 ± 11.80 | 53.24 ± 9.12 | 35.76 ± 2.76 | 22.86 ± 2.27 | 9.82 ± 1.26 | 23.21 ± 2.32 | 17.14 ± 2.36 |
| **PVC4** | 85.39 ± 14.20 | 46.09 ± 5.30 | 30.63 ± 3.24 | 25.23 ± 2.36 | 11.59 ± 1.15 | 21.70 ± 2.62 | 20.23 ± 3.39 |
| **PVC5** | 89.62 ± 7.40 | 46.17 ± 7.21 | 31.97 ± 2.66 | 25.23 ± 3.98 | 9.77 ± 1.35 | 20.79 ± 3.39 | 18.26 ± 2.32 |
| **PVC6** | 104.81 ± 14.36 | 51.62 ± 7.60 | 31.25 ± 3.55 | 23.43 ± 2.07 | 9.53 ± 1.44 | 23.62 ± 3.03 | 17.03 ± 1.68 |
| **PVC7** | 98.98 ± 15.03 | 57.03 ± 8.76 | 33.85 ± 3.82 | 23.13 ± 3.65 | 10.31 ± 0.97 | 22.04 ± 3.23 | 16.33 ± 2.06 |
| **PVC8** | 93.16 ± 7.66 | 45.70 ± 7.09 | 31.66 ± 4.88 | 25.93 ± 4.16 | 10.18 ± 1.41 | 21.15 ± 2.31 | 18.99 ± 2.86 |
| **PVC9** | 86.02 ± 14.76 | 52.80 ± 4.78 | 33.95 ± 3.73 | 23.26 ± 3.96 | 11.82 ± 1.81 | 24.40 ± 2.05 | 19.64 ± 2.66 |
|  | **Concentration (Mean ± SD, ng/kg of lipid)** | | | | | | |
| **Sample** | **PCB 167** | **PCB 169** | **PCB 170** | **PCB 180** | **PCB 189** | **PCB 195** | **PCB 209** |
| **W1** | N.D. | N.D. | N.D. | 32.80 ± 3.62 | 11.58 ± 0.93 | 11.24 ± 1.00 | 6.00 ± 0.70 |
| **W2** | N.D. | N.D. | N.D. | 28.03 ± 3.20 | 12.25 ± 1.00 | 12.21 ± 1.33 | 6.45 ± 0.95 |
| **W3** | N.D. | N.D. | N.D. | 27.87 ± 3.95 | 11.94 ± 1.68 | 10.54 ± 1.20 | 7.44 ± 0.58 |
| **W4** | N.D. | N.D. | N.D. | 33.36 ± 3.41 | 10.95 ± 0.91 | 13.17 ± 1.79 | 6.98 ± 0.95 |
| **W5** | N.D. | N.D. | N.D. | 41.83 ± 6.40 | 9.64 ± 1.12 | 14.01 ± 1.15 | 5.73 ± 0.61 |
| **W6** | N.D. | N.D. | N.D. | 34.73 ± 4.58 | 10.13 ± 1.61 | 11.41 ± 1.08 | 4.94 ± 0.66 |
| **W7** | N.D. | N.D. | N.D. | 40.01 ± 4.91 | 12.50 ± 1.16 | 14.02 ± 1.42 | 5.53 ± 0.67 |
| **W8** | N.D. | N.D. | N.D. | 30.76 ± 5.12 | 9.68 ± 0.92 | 13.01 ± 1.78 | 6.80 ± 0.99 |
| **W9** | N.D. | N.D. | N.D. | 25.37 ± 3.79 | 10.75 ± 1.73 | 12.34 ± 1.48 | 5.34 ± 0.43 |
| **PE1** | 12.37 ± 1.77 | 11.46 ± 1.14 | N.D. | 58.79 ± 7.37 | 30.76 ± 2.49 | 11.95 ± 1.29 | 11.30 ± 1.91 |
| **PE2** | 13.59 ± 1.21 | 13.76 ± 1.49 | N.D. | 57.45 ± 9.62 | 33.42 ± 3.58 | 12.34 ± 1.70 | 12.49 ± 1.74 |
| **PE3** | 8.87 ± 0.90 | 11.60 ± 1.44 | N.D. | 58.21 ± 7.47 | 32.04 ± 4.23 | 9.21 ± 0.87 | 11.21 ± 1.14 |
| **PE4** | 11.64 ± 1.55 | 8.42 ± 1.23 | N.D. | 73.09 ± 6.84 | 23.00 ± 2.78 | 13.49 ± 1.77 | 14.12 ± 1.78 |
| **PE5** | 15.64 ± 1.26 | 11.73 ± 1.12 | N.D. | 71.63 ± 10.39 | 27.30 ± 2.57 | 9.15 ± 1.13 | 7.62 ± 1.27 |
| **PE6** | 16.14 ± 2.21 | 13.86 ± 1.51 | N.D. | 63.79 ± 5.06 | 40.79 ± 6.41 | 10.39 ± 1.74 | 8.00 ± 0.65 |
| **PE7** | 13.67 ± 1.92 | 12.54 ± 1.01 | N.D. | 42.41 ± 7.13 | 24.18 ± 3.50 | 14.75 ± 2.53 | 13.43 ± 2.18 |
| **PE8** | 11.53 ± 1.47 | 7.74 ± 0.84 | N.D. | 50.26 ± 8.36 | 26.77 ± 2.23 | 8.94 ± 0.82 | 12.20 ± 1.38 |
| **PE9** | 13.70 ± 1.89 | 13.16 ± 1.23 | N.D. | 59.53 ± 5.54 | 35.40 ± 4.95 | 15.89 ± 2.29 | 10.26 ± 0.81 |
| **PS1** | 20.90 ± 2.75 | 8.96 ± 1.13 | 14.19 ± 1.12 | 66.58 ± 7.59 | 41.71 ± 4.75 | 23.84 ± 2.58 | 24.85 ± 3.73 |
| **PS2** | 20.80 ± 1.70 | 6.98 ± 1.17 | 11.45 ± 1.12 | 60.30 ± 9.39 | 41.71 ± 5.43 | 22.39 ± 2.43 | 23.01 ± 1.88 |
| **PS3** | 22.13 ± 1.75 | 7.18 ± 0.85 | 8.91 ± 1.30 | 57.71 ± 9.01 | 36.60 ± 3.57 | 17.38 ± 2.60 | 29.92 ± 2.54 |
| **PS4** | 24.45 ± 3.23 | 8.98 ± 1.24 | 9.15 ± 1.05 | 57.15 ± 9.53 | 41.08 ± 5.17 | 25.32 ± 2.88 | 17.71 ± 2.18 |
| **PS5** | 16.30 ± 2.69 | 8.16 ± 1.31 | 11.47 ± 1.80 | 77.23 ± 7.18 | 43.46 ± 3.58 | 27.58 ± 4.26 | 24.66 ± 3.23 |
| **PS6** | 27.19 ± 3.68 | 6.69 ± 0.87 | 10.50 ± 0.98 | 52.89 ± 7.52 | 51.99 ± 5.67 | 24.00 ± 3.84 | 27.90 ± 4.61 |
| **PS7** | 19.44 ± 2.42 | 8.06 ± 1.17 | 12.93 ± 1.27 | 53.39 ± 4.88 | 42.72 ± 6.11 | 29.78 ± 4.69 | 21.81 ± 1.80 |
| **PS8** | 23.58 ± 2.07 | 8.06 ± 0.84 | 15.03 ± 1.18 | 51.45 ± 4.28 | 32.14 ± 2.53 | 20.85 ± 3.34 | 20.28 ± 3.40 |
| **PS9** | 26.87 ± 2.05 | 5.85 ± 0.94 | 12.00 ± 1.44 | 72.38 ± 7.85 | 37.30 ± 6.38 | 23.83 ± 2.70 | 25.71 ± 3.08 |
| **PVC1** | 21.49 ± 2.83 | 23.67 ± 2.08 | 11.16 ± 1.85 | 110.97 ± 14.98 | 91.56 ± 12.92 | 12.33 ± 2.06 | 58.29 ± 9.33 |
| **PVC2** | 19.88 ± 2.25 | 25.47 ± 3.08 | 11.60 ± 1.64 | 91.77 ± 12.96 | 97.76 ± 9.93 | 10.92 ± 1.05 | 60.94 ± 7.48 |
| **PVC3** | 21.41 ± 3.17 | 22.91 ± 3.00 | 9.75 ± 1.12 | 123.22 ± 15.02 | 96.88 ± 8.25 | 12.80 ± 1.78 | 59.33 ± 8.10 |
| **PVC4** | 20.03 ± 1.77 | 24.35 ± 2.23 | 11.70 ± 1.61 | 104.31 ± 9.30 | 87.05 ± 10.28 | 11.64 ± 1.73 | 63.89 ± 5.67 |
| **PVC5** | 22.16 ± 1.92 | 26.09 ± 2.03 | 10.77 ± 1.34 | 105.53 ± 11.01 | 83.19 ± 9.84 | 13.07 ± 1.05 | 54.40 ± 4.96 |
| **PVC6** | 21.65 ± 2.32 | 20.97 ± 2.91 | 11.32 ± 1.24 | 104.44 ± 16.84 | 84.03 ± 9.72 | 11.07 ± 1.82 | 53.55 ± 6.57 |
| **PVC7** | 21.31 ± 1.89 | 21.02 ± 3.07 | 10.79 ± 0.96 | 107.51 ± 8.90 | 92.15 ± 8.34 | 13.49 ± 1.09 | 53.51 ± 7.25 |
| **PVC8** | 20.57 ± 2.83 | 24.91 ± 2.93 | 11.71 ± 1.49 | 113.83 ± 9.36 | 98.08 ± 11.94 | 13.29 ± 2.17 | 61.84 ± 8.54 |
| **PVC9** | 23.42 ± 3.67 | 23.38 ± 3.43 | 11.12 ± 0.93 | 122.40 ± 17.77 | 101.23 ± 16.56 | 10.95 ± 0.84 | 54.94 ± 8.83 |
|  | **Concentration (Mean ± SD, ng/kg of lipid)** | | | | | | |
| **Sample** | **123478-HxCDD** | **1234678-HpCDD** | **2378-TCDF** | **12378-PeCDF** | **23478-PeCDF** | **123478-HxCDF** | **123678-HxCDF** |
| **W1** | N.D. | N.D. | N.D. | N.D. | N.D. | N.D. | N.D. |
| **W2** | N.D. | N.D. | N.D. | N.D. | N.D. | N.D. | N.D. |
| **W3** | N.D. | N.D. | N.D. | N.D. | N.D. | N.D. | N.D. |
| **W4** | N.D. | N.D. | N.D. | N.D. | N.D. | N.D. | N.D. |
| **W5** | N.D. | N.D. | N.D. | N.D. | N.D. | N.D. | N.D. |
| **W6** | N.D. | N.D. | N.D. | N.D. | N.D. | N.D. | N.D. |
| **W7** | N.D. | N.D. | N.D. | N.D. | N.D. | N.D. | N.D. |
| **W8** | N.D. | N.D. | N.D. | N.D. | N.D. | N.D. | N.D. |
| **W9** | N.D. | N.D. | N.D. | N.D. | N.D. | N.D. | N.D. |
| **PE1** | N.D. | N.D. | 0.28 ± 0.02 | 0.23 ± 0.02 | N.D. | N.D. | 0.29 ± 0.04 |
| **PE2** | N.D. | N.D. | 0.32 ± 0.03 | 0.22 ± 0.02 | N.D. | N.D. | 0.28 ± 0.05 |
| **PE3** | N.D. | N.D. | 0.33 ± 0.04 | 0.19 ± 0.02 | N.D. | N.D. | 0.22 ± 0.02 |
| **PE4** | N.D. | N.D. | 0.28 ± 0.03 | 0.23 ± 0.03 | N.D. | N.D. | 0.28 ± 0.04 |
| **PE5** | N.D. | N.D. | 0.20 ± 0.02 | 0.31 ± 0.02 | N.D. | N.D. | 0.31 ± 0.04 |
| **PE6** | N.D. | N.D. | 0.24 ± 0.03 | 0.17 ± 0.02 | N.D. | N.D. | 0.34 ± 0.04 |
| **PE7** | N.D. | N.D. | 0.32 ± 0.03 | 0.24 ± 0.04 | N.D. | N.D. | 0.37 ± 0.05 |
| **PE8** | N.D. | N.D. | 0.33 ± 0.05 | 0.30 ± 0.04 | N.D. | N.D. | 0.28 ± 0.04 |
| **PE9** | N.D. | N.D. | 0.21 ± 0.02 | 0.23 ± 0.03 | N.D. | N.D. | 0.22 ± 0.04 |
| **PS1** | 0.63 ± 0.06 | 0.93 ± 0.11 | 0.93 ± 0.09 | 0.38 ± 0.06 | N.D. | 0.58 ± 0.10 | 0.61 ± 0.06 |
| **PS2** | 0.72 ± 0.11 | 0.34 ± 0.03 | 1.18 ± 0.10 | 0.90 ± 0.08 | N.D. | 0.33 ± 0.03 | 0.39 ± 0.06 |
| **PS3** | 0.93 ± 0.13 | 0.44 ± 0.05 | 1.50 ± 0.25 | 1.04 ± 0.09 | N.D. | 0.40 ± 0.03 | 0.38 ± 0.06 |
| **PS4** | 0.55 ± 0.06 | 0.33 ± 0.03 | 1.19 ± 0.13 | 1.11 ± 0.19 | N.D. | 0.33 ± 0.04 | 0.47 ± 0.05 |
| **PS5** | 0.57 ± 0.10 | 0.38 ± 0.03 | 1.12 ± 0.09 | 0.97 ± 0.09 | N.D. | 0.36 ± 0.05 | 0.43 ± 0.04 |
| **PS6** | 0.80 ± 0.10 | 0.29 ± 0.03 | 1.58 ± 0.14 | 1.11 ± 0.13 | N.D. | 0.38 ± 0.05 | 0.47 ± 0.08 |
| **PS7** | 0.73 ± 0.08 | 0.27 ± 0.04 | 1.20 ± 0.18 | 1.17 ± 0.16 | N.D. | 0.30 ± 0.03 | 0.48 ± 0.06 |
| **PS8** | 0.60 ± 0.05 | 0.44 ± 0.04 | 1.27 ± 0.13 | 0.84 ± 0.08 | N.D. | 0.43 ± 0.06 | 0.34 ± 0.05 |
| **PS9** | 0.71 ± 0.11 | 0.34 ± 0.03 | 1.50 ± 0.18 | 0.93 ± 0.10 | N.D. | 0.37 ± 0.03 | 0.52 ± 0.08 |
| **PVC1** | 1.56 ± 0.15 | 1.07 ± 0.13 | 3.98 ± 0.39 | 4.99 ± 0.72 | 5.01 ± 0.63 | 3.21 ± 0.32 | 4.04 ± 0.45 |
| **PVC2** | 1.50 ± 0.15 | 0.96 ± 0.09 | 1.18 ± 0.19 | 3.16 ± 0.35 | 5.26 ± 0.81 | 2.63 ± 0.30 | 2.66 ± 0.23 |
| **PVC3** | 1.63 ± 0.26 | 0.97 ± 0.11 | 4.20 ± 0.57 | 4.80 ± 0.69 | 4.63 ± 0.44 | 3.34 ± 0.35 | 3.61 ± 0.57 |
| **PVC4** | 1.62 ± 0.26 | 1.08 ± 0.17 | 4.25 ± 0.39 | 4.58 ± 0.35 | 5.32 ± 0.79 | 2.95 ± 0.30 | 3.77 ± 0.32 |
| **PVC5** | 1.59 ± 0.24 | 0.97 ± 0.08 | 4.44 ± 0.63 | 5.59 ± 0.84 | 5.03 ± 0.51 | 2.81 ± 0.36 | 4.35 ± 0.71 |
| **PVC6** | 1.60 ± 0.20 | 1.03 ± 0.13 | 3.98 ± 0.51 | 4.57 ± 0.67 | 5.59 ± 0.45 | 2.81 ± 0.44 | 4.24 ± 0.47 |
| **PVC7** | 1.64 ± 0.20 | 1.08 ± 0.16 | 4.15 ± 0.54 | 5.37 ± 0.53 | 5.32 ± 0.52 | 3.23 ± 0.34 | 4.39 ± 0.51 |
| **PVC8** | 1.40 ± 0.15 | 1.12 ± 0.17 | 4.14 ± 0.41 | 4.68 ± 0.51 | 4.74 ± 0.38 | 3.50 ± 0.36 | 4.01 ± 0.61 |
| **PVC9** | 1.40 ± 0.18 | 1.04 ± 0.12 | 4.04 ± 0.39 | 4.53 ± 0.63 | 4.41 ± 0.57 | 3.05 ± 0.28 | 3.59 ± 0.52 |

|  | **Concentration (Mean ± SD, ng/kg of lipid)** | | | | | | | |
| --- | --- | --- | --- | --- | --- | --- | --- | --- |
| **Sample** | **123789-HxCDF** | **234678-HxCDF** | **1234678-HpCDF** | **1234789-HpCDF** | **OCDF** | **Total PCBs** | **Total PCDD/Fs** | **TEQ** |
| **W1** | N.D. | N.D. | N.D. | N.D. | N.D. | 143.56 ± 14.63 | N.D. | 0.0036 ± 0.0002 |
| **W2** | N.D. | N.D. | N.D. | N.D. | N.D. | 154.81 ± 14.07 | N.D. | 0.0031 ± 0.0001 |
| **W3** | N.D. | N.D. | N.D. | N.D. | N.D. | 153.84 ± 23.00 | N.D. | 0.0038 ± 0.0002 |
| **W4** | N.D. | N.D. | N.D. | N.D. | N.D. | 158.42 ± 25.86 | N.D. | 0.0046 ± 0.0002 |
| **W5** | N.D. | N.D. | N.D. | N.D. | N.D. | 165.19 ± 19.28 | N.D. | 0.0038 ± 0.0002 |
| **W6** | N.D. | N.D. | N.D. | N.D. | N.D. | 158.99 ± 15.39 | N.D. | 0.0044 ± 0.0002 |
| **W7** | N.D. | N.D. | N.D. | N.D. | N.D. | 152.06 ± 23.03 | N.D. | 0.0036 ± 0.0002 |
| **W8** | N.D. | N.D. | N.D. | N.D. | N.D. | 146.01 ± 12.11 | N.D. | 0.0040 ± 0.0002 |
| **W9** | N.D. | N.D. | N.D. | N.D. | N.D. | 138.60 ± 13.83 | N.D. | 0.0035 ± 0.0002 |
| **PE1** | N.D. | 0.28 ± 0.04 | N.D. | N.D. | N.D. | 463.16 ± 36.43 | 1.08 ± 0.14 | 0.4321 ± 0.0187 |
| **PE2** | N.D. | 0.30 ± 0.04 | N.D. | N.D. | N.D. | 461.60 ± 50.89 | 1.12 ± 0.12 | 0.4414 ± 0.0179 |
| **PE3** | N.D. | 0.28 ± 0.03 | N.D. | N.D. | N.D. | 451.04 ± 59.89 | 1.02 ± 0.13 | 0.4266 ± 0.0261 |
| **PE4** | N.D. | 0.19 ± 0.02 | N.D. | N.D. | N.D. | 468.39 ± 64.40 | 0.98 ± 0.12 | 0.4496 ± 0.0218 |
| **PE5** | N.D. | 0.31 ± 0.04 | N.D. | N.D. | N.D. | 451.24 ± 62.07 | 1.13 ± 0.17 | 0.5094 ± 0.0163 |
| **PE6** | N.D. | 0.19 ± 0.02 | N.D. | N.D. | N.D. | 481.24 ± 48.42 | 0.94 ± 0.13 | 0.4575 ± 0.0157 |
| **PE7** | N.D. | 0.23 ± 0.02 | N.D. | N.D. | N.D. | 454.31 ± 61.66 | 1.16 ± 0.16 | 0.4839 ± 0.0242 |
| **PE8** | N.D. | 0.32 ± 0.03 | N.D. | N.D. | N.D. | 458.73 ± 58.47 | 1.23 ± 0.19 | 0.4819 ± 0.0228 |
| **PE9** | N.D. | 0.23 ± 0.03 | N.D. | N.D. | N.D. | 493.92 ± 56.03 | 0.89 ± 0.11 | 0.4267 ± 0.0205 |
| **PS1** | N.D. | 0.63 ± 0.08 | 1.19 ± 0.12 | N.D. | N.D. | 719.62 ± 91.91 | 5.88 ± 0.93 | 1.7417 ± 0.0924 |
| **PS2** | N.D. | 0.57 ± 0.07 | 0.27 ± 0.02 | N.D. | N.D. | 781.37 ± 97.53 | 4.70 ± 0.41 | 2.0700 ± 0.0581 |
| **PS3** | N.D. | 0.73 ± 0.10 | 0.25 ± 0.03 | N.D. | N.D. | 829.12 ± 100.15 | 5.67 ± 0.87 | 1.8681 ± 0.0770 |
| **PS4** | N.D. | 0.56 ± 0.06 | 0.36 ± 0.03 | N.D. | N.D. | 794.57 ± 65.06 | 4.90 ± 0.70 | 1.8181 ± 0.0568 |
| **PS5** | N.D. | 0.69 ± 0.07 | 0.29 ± 0.05 | N.D. | N.D. | 856.39 ± 142.98 | 4.81 ± 0.55 | 2.5340 ± 0.0552 |
| **PS6** | N.D. | 0.72 ± 0.07 | 0.35 ± 0.05 | N.D. | N.D. | 783.88 ± 123.75 | 5.70 ± 0.94 | 2.3376 ± 0.0641 |
| **PS7** | N.D. | 0.49 ± 0.05 | 0.34 ± 0.05 | N.D. | N.D. | 812.38 ± 101.05 | 4.98 ± 0.63 | 2.1107 ± 0.0649 |
| **PS8** | N.D. | 0.65 ± 0.08 | 0.33 ± 0.03 | N.D. | N.D. | 846.74 ± 95.85 | 4.90 ± 0.79 | 2.6262 ± 0.0684 |
| **PS9** | N.D. | 0.64 ± 0.06 | 0.31 ± 0.03 | N.D. | N.D. | 825.46 ± 112.65 | 5.32 ± 0.90 | 2.4228 ± 0.0606 |
| **PVC1** | 0.74 ± 0.11 | 2.51 ± 0.40 | 4.12 ± 0.63 | 2.26 ± 0.33 | 1.15 ± 0.10 | 1007.62 ± 142.24 | 34.64 ± 2.69 | 4.5462 ± 0.1915 |
| **PVC2** | 1.07 ± 0.08 | 3.89 ± 0.48 | 3.53 ± 0.55 | 1.06 ± 0.18 | 0.99 ± 0.17 | 926.44 ± 78.12 | 27.89 ± 2.63 | 4.5010 ± 0.1763 |
| **PVC3** | 0.68 ± 0.07 | 2.30 ± 0.26 | 4.18 ± 0.39 | 2.13 ± 0.32 | 1.02 ± 0.14 | 1008.31 ± 160.01 | 33.49 ± 3.56 | 4.3392 ± 0.1781 |
| **PVC4** | 0.66 ± 0.11 | 2.27 ± 0.26 | 4.60 ± 0.56 | 2.52 ± 0.23 | 1.03 ± 0.09 | 995.06 ± 155.83 | 34.65 ± 5.43 | 4.4749 ± 0.2194 |
| **PVC5** | 0.66 ± 0.09 | 2.59 ± 0.32 | 3.76 ± 0.61 | 2.20 ± 0.25 | 1.22 ± 0.20 | 947.49 ± 151.97 | 35.21 ± 5.52 | 4.4581 ± 0.2267 |
| **PVC6** | 0.66 ± 0.06 | 2.46 ± 0.41 | 4.28 ± 0.44 | 2.05 ± 0.27 | 1.07 ± 0.08 | 997.58 ± 123.11 | 34.34 ± 3.04 | 4.3382 ± 0.1709 |
| **PVC7** | 0.77 ± 0.10 | 2.43 ± 0.21 | 4.63 ± 0.42 | 2.50 ± 0.33 | 1.06 ± 0.12 | 1006.75 ± 122.53 | 36.57 ± 4.31 | 4.5313 ± 0.2058 |
| **PVC8** | 0.76 ± 0.08 | 2.79 ± 0.47 | 4.17 ± 0.44 | 2.47 ± 0.39 | 1.10 ± 0.09 | 1010.67 ± 147.15 | 34.88 ± 4.47 | 4.6666 ± 0.2284 |
| **PVC9** | 0.70 ± 0.07 | 2.76 ± 0.42 | 4.41 ± 0.59 | 2.49 ± 0.21 | 1.05 ± 0.12 | 1002.85 ± 141.42 | 33.47 ± 4.02 | 4.3139 ± 0.2170 |

N.D.: not detected. W: wood; PE: wood + polyethylene; PS: wood + polystyrene; PVC: wood + polyvinyl chloride.
