# Supplementary material for: Effect of plastic composition in the combustion material on the Persistent Organic Pollutant content in smoked chicken meat
Source: PLoS One. 2026 Jun 3;21(6):e0350345. doi: 10.1371/journal.pone.0350345 (PMC13232828; doi:10.1371/journal.pone.0350345)
Supplement: S2 Table — Statistical significance was determined at p < 0.05, indicating that the corresponding PCB or PCDD/F congener exhibited significant differences in concentration between the compared fuel groups. Adjusted p-values were obtained using the Benjamini–Hochberg (BH) method. Values shown in bold indicate statistically significant differences. NA indicates that the p-value could not be computed due to tied or zero differences in paired data. (DOCX) [file pone.0350345.s005.docx]

**Table S2. Pairwise comparison results from Wilcoxon signed-rank tests with Benjamini–Hochberg correction for selected PCBs and PCDD/Fs in smoked chicken meat samples across different fuel types. Statistical significance was determined at p < 0.05, indicating that the corresponding PCB or PCDD/F congener exhibited significant differences in concentration between the compared fuel groups. Adjusted p-values were obtained using the Benjamini–Hochberg (BH) method. Values shown in bold indicate statistically significant differences. NA indicates that the p-value could not be computed due to tied or zero differences in paired data.**

| **Group1** | | **Group2** | | **PCB 66** | | **PCB 28** | | **PCB 101** | | **PCB 153** | | | | | **PCB 180** | | | | **PCB 189** | | | **PCB 195** | | | |  |  |  |  |  |  |
| --- | --- | --- | --- | --- | --- | --- | --- | --- | --- | --- | --- | --- | --- | --- | --- | --- | --- | --- | --- | --- | --- | --- | --- | --- | --- | --- | --- | --- | --- | --- | --- |
| W | | PE | | NA | | **0.00**5 | | **0.00**5 | | **0.00**5 | | | | | **0.00**5 | | | | **0.00**4 | | | 0.684 | | | |  |  |  |  |  |  |
|  |  | PS | | **0.004** | | **0.005** | | 1 | | **0.005** | | | | | **0.005** | | | | **0.004** | | | **0.008** | | | |  |  |  |  |  |  |
|  |  | PVC | | **0.004** | | **0.005** | | **0.005** | | **0.005** | | | | | **0.005** | | | | **0.004** | | | 0.639 | | | |  |  |  |  |  |  |
| PE | | PS | | **0.00**4 | | **0.00**5 | | **0.00**5 | | 1 | | | | | 0.496 | | | | **0.00**4 | | | **0.00**8 | | | |  |  |  |  |  |  |
|  |  | PVC | | **0.004** | | **0.005** | | **0.005** | | **0.005** | | | | | **0.005** | | | | **0.004** | | | 0.91 | | | |  |  |  |  |  |  |
| PS | | PVC | | **0.004** | | 0.82 | | **0.005** | | **0.005** | | | | | **0.005** | | | | **0.004** | | | **0.008** | | | |  |  |  |  |  |  |
|  |  | |  | |  | |  | |  | | |  | |  | | |  | | | |  | | |  | | |  | |  | |  |
| **Group1** | **Group2** | | **23478-PeCDF** | | **2378-TCDF** | | **OCDF** | | **1234678-HpCDD** | | **1234678-HpCDF** | | **123478-HxCDD** | | | **123478-HxCDF** | | **1234789-HpCDF** | | **123678-HxCDF** | | | **12378-PeCDF** | | **123789-HxCDF** | | | **234678-HxCDF** | |  |  |
| W | PE | | NA | | **0.00**9 | | NA | | NA | | NA | | NA | | | NA | | NA | | **0.00**4 | | | **0.00**4 | | NA | | | **0.00**4 | |  |  |
|  | PS | | NA | | **0.006** | | NA | | **0.004** | | **0.004** | | **0.004** | | | **0.004** | | NA | | **0.004** | | | **0.004** | | NA | | | **0.004** | |  |  |
|  | PVC | | **0.004** | | **0.006** | | **0.004** | | **0.004** | | **0.004** | | **0.004** | | | **0.004** | | **0.004** | | **0.004** | | | **0.004** | | **0.009** | | | **0.004** | |  |  |
| PE | PS | | NA | | **0.00**6 | | NA | | **0.00**4 | | **0.00**4 | | **0.00**4 | | | **0.00**4 | | NA | | **0.00**4 | | | **0.00**4 | | NA | | | **0.00**4 | |  |  |
|  | PVC | | **0.004** | | **0.006** | | **0.004** | | **0.004** | | **0.004** | | **0.004** | | | **0.004** | | **0.004** | | **0.004** | | | **0.004** | | **0.009** | | | **0.004** | |  |  |
| PS | PVC | | **0.004** | | **0.009** | | **0.004** | | **0.004** | | **0.004** | | **0.004** | | | **0.004** | | **0.004** | | **0.004** | | | **0.004** | | **0.009** | | | **0.004** | |  |  |

NA indicates that the p-value could not be computed due to tied or zero differences in paired data.
