# Supplementary material for: Effect of plastic composition in the combustion material on the Persistent Organic Pollutant content in smoked chicken meat
Source: PLoS One. 2026 Jun 3;21(6):e0350345. doi: 10.1371/journal.pone.0350345 (PMC13232828; doi:10.1371/journal.pone.0350345)
Supplement: S3 Table — (DOCX) [file pone.0350345.s006.docx]

**Table S3. PAHs concentrations of the samples**

| **Sample** | **Concentration (Mean ± SD, µg/kg of lipid)** | | | | | |
| --- | --- | --- | --- | --- | --- | --- |
|  | **Naphthalene** | **Acenaphthylene** | **Acenaphthene** | **Fluorene** | **Phenanthrene** | **Fluoranthene** |
| **W1** | 157.32 ± 12.02 | 1.12 ± 0.15 | 2.30 ± 0.33 | 16.60 ± 2.58 | 208.34 ± 30.57 | N.D. |
| **W2** | 129.23 ± 19.77 | 0.44 ± 0.05 | 1.20 ± 0.19 | 23.70 ± 2.06 | 210.77 ± 23.79 | N.D. |
| **W3** | 164.37 ± 28.11 | 0.93 ± 0.12 | 2.33 ± 0.40 | 23.45 ± 2.08 | 262.63 ± 31.87 | N.D. |
| **W4** | 141.99 ± 12.26 | 0.71 ± 0.11 | 2.09 ± 0.25 | 24.30 ± 4.02 | 235.19 ± 29.85 | N.D. |
| **W5** | 160.88 ± 15.28 | 0.72 ± 0.11 | 1.90 ± 0.24 | 23.83 ± 4.09 | 271.96 ± 26.28 | N.D. |
| **W6** | 184.97 ± 24.27 | 0.81 ± 0.09 | 2.05 ± 0.32 | 25.77 ± 4.09 | 232.84 ± 25.68 | N.D. |
| **W7** | 162.69 ± 23.20 | 0.83 ± 0.06 | 2.30 ± 0.18 | 26.72 ± 3.98 | 208.10 ± 28.47 | N.D. |
| **W8** | 152.50 ± 21.62 | 0.97 ± 0.16 | 1.65 ± 0.17 | 23.85 ± 3.53 | 243.06 ± 38.62 | N.D. |
| **W9** | 200.92 ± 21.71 | 0.88 ± 0.09 | 2.13 ± 0.30 | 19.79 ± 3.12 | 268.36 ± 35.07 | N.D. |
| **PE1** | 681.62 ± 73.91 | 6.72 ± 1.11 | 9.70 ± 1.06 | 147.58 ± 12.07 | 520.13 ± 57.26 | 14.50 ± 1.30 |
| **PE2** | 904.85 ± 145.97 | 6.22 ± 0.48 | 12.51 ± 2.10 | 112.94 ± 12.07 | 536.34 ± 91.90 | 21.80 ± 2.69 |
| **PE3** | 809.30 ± 110.28 | 7.11 ± 0.91 | 11.70 ± 1.74 | 143.10 ± 22.52 | 532.06 ± 74.25 | 23.59 ± 3.97 |
| **PE4** | 773.65 ± 125.65 | 7.67 ± 0.61 | 9.26 ± 1.02 | 134.93 ± 11.43 | 579.96 ± 76.81 | 22.03 ± 1.96 |
| **PE5** | 890.48 ± 151.68 | 6.58 ± 1.11 | 9.03 ± 1.29 | 124.35 ± 18.48 | 566.57 ± 43.40 | 18.75 ± 2.39 |
| **PE6** | 891.92 ± 101.71 | 7.42 ± 1.27 | 10.88 ± 1.75 | 141.53 ± 15.02 | 545.79 ± 48.04 | 24.14 ± 3.67 |
| **PE7** | 690.26 ± 112.68 | 7.86 ± 0.61 | 8.71 ± 1.28 | 173.20 ± 28.60 | 554.52 ± 66.64 | 20.90 ± 2.53 |
| **PE8** | 672.17 ± 76.03 | 8.85 ± 1.49 | 11.16 ± 0.94 | 159.82 ± 12.41 | 538.11 ± 49.89 | 18.74 ± 2.64 |
| **PE9** | 812.95 ± 109.98 | 7.49 ± 1.11 | 11.47 ± 1.42 | 131.71 ± 11.53 | 603.93 ± 64.93 | 18.53 ± 2.33 |
| **PS1** | 193.87 ± 19.22 | 3.59 ± 0.39 | 4.84 ± 0.76 | 38.20 ± 4.82 | 597.89 ± 57.09 | 69.80 ± 11.79 |
| **PS2** | 198.29 ± 29.12 | 3.91 ± 0.50 | 5.64 ± 0.96 | 51.10 ± 8.34 | 576.54 ± 72.76 | 58.70 ± 5.71 |
| **PS3** | 261.04 ± 36.82 | 3.55 ± 0.42 | 6.01 ± 0.56 | 54.00 ± 6.73 | 582.55 ± 65.88 | 70.50 ± 8.17 |
| **PS4** | 225.20 ± 29.36 | 4.15 ± 0.41 | 5.97 ± 0.91 | 42.87 ± 7.11 | 697.67 ± 112.26 | 65.30 ± 5.63 |
| **PS5** | 242.71 ± 31.84 | 3.74 ± 0.29 | 5.67 ± 0.90 | 53.23 ± 6.51 | 761.71 ± 68.48 | 76.90 ± 8.03 |
| **PS6** | 195.94 ± 17.08 | 3.53 ± 0.47 | 5.34 ± 0.57 | 53.72 ± 6.08 | 720.98 ± 83.83 | 59.20 ± 6.12 |
| **PS7** | 227.10 ± 30.04 | 3.72 ± 0.62 | 5.29 ± 0.47 | 41.73 ± 5.29 | 615.75 ± 68.30 | 72.20 ± 7.24 |
| **PS8** | 196.31 ± 18.35 | 4.94 ± 0.51 | 6.45 ± 0.88 | 50.82 ± 6.59 | 772.32 ± 99.74 | 80.00 ± 9.26 |
| **PS9** | 183.94 ± 22.60 | 3.78 ± 0.54 | 5.13 ± 0.48 | 59.71 ± 7.23 | 596.37 ± 47.73 | 69.90 ± 9.89 |
| **PVC1** | 417.45 ± 63.32 | 4.61 ± 0.43 | 6.78 ± 0.81 | 55.50 ± 6.19 | 607.64 ± 88.04 | 31.00 ± 5.06 |
| **PVC2** | 490.48 ± 49.36 | 4.56 ± 0.59 | 7.40 ± 0.95 | 68.00 ± 6.28 | 732.91 ± 98.31 | 33.10 ± 2.79 |
| **PVC3** | 407.80 ± 65.55 | 5.15 ± 0.82 | 8.86 ± 0.82 | 66.75 ± 9.36 | 721.87 ± 119.18 | 31.00 ± 2.38 |
| **PVC4** | 393.97 ± 59.97 | 4.31 ± 0.46 | 8.30 ± 0.84 | 80.30 ± 6.30 | 581.40 ± 64.35 | 39.05 ± 4.49 |
| **PVC5** | 463.20 ± 54.82 | 5.96 ± 0.66 | 8.24 ± 1.05 | 78.87 ± 13.48 | 768.29 ± 80.62 | 39.72 ± 5.63 |
| **PVC6** | 464.41 ± 35.44 | 4.85 ± 0.60 | 8.49 ± 1.33 | 81.59 ± 7.94 | 612.16 ± 95.14 | 37.98 ± 4.82 |
| **PVC7** | 382.80 ± 37.86 | 5.93 ± 0.94 | 6.05 ± 0.73 | 79.23 ± 13.37 | 783.60 ± 98.82 | 33.69 ± 5.73 |
| **PVC8** | 429.54 ± 44.18 | 4.39 ± 0.44 | 8.31 ± 0.85 | 60.29 ± 9.33 | 811.65 ± 93.09 | 38.29 ± 6.42 |
| **PVC9** | 380.85 ± 65.15 | 4.70 ± 0.44 | 7.12 ± 1.01 | 65.68 ± 8.67 | 677.08 ± 78.58 | 37.25 ± 6.36 |
| **Sample** | **Concentration (Mean ± SD, µg/kg of lipid)** | | | | | |
|  | **Pyrene** | **Benzo[a]anthracene** | **Chrysene** | **Benzo[b]fluoranthene** | **Benzo[k]fluoranthene** | **Benzo[e]pyrene** |
| **W1** | 16.60 ± 1.56 | 19.10 ± 1.78 | 38.20 ± 3.25 | 19.50 ± 2.66 | 5.32 ± 0.48 | N.D. |
| **W2** | 15.70 ± 1.74 | 17.30 ± 1.54 | 44.25 ± 5.59 | 21.80 ± 3.75 | 6.81 ± 0.92 | N.D. |
| **W3** | 14.60 ± 1.98 | 19.28 ± 1.69 | 37.63 ± 4.12 | 27.49 ± 2.47 | 4.88 ± 0.73 | N.D. |
| **W4** | 19.04 ± 2.22 | 20.65 ± 1.98 | 43.10 ± 6.24 | 25.96 ± 2.50 | 6.54 ± 0.62 | N.D. |
| **W5** | 20.10 ± 2.13 | 19.31 ± 2.72 | 38.79 ± 4.63 | 19.81 ± 3.38 | 6.44 ± 0.85 | N.D. |
| **W6** | 15.15 ± 2.38 | 21.44 ± 2.07 | 40.32 ± 6.42 | 23.94 ± 3.64 | 6.81 ± 1.00 | N.D. |
| **W7** | 19.27 ± 2.91 | 17.25 ± 1.42 | 42.79 ± 4.86 | 20.40 ± 2.59 | 5.11 ± 0.77 | N.D. |
| **W8** | 18.82 ± 2.53 | 22.09 ± 2.75 | 49.31 ± 5.24 | 25.74 ± 4.37 | 5.82 ± 0.69 | N.D. |
| **W9** | 13.98 ± 1.79 | 24.02 ± 2.04 | 48.80 ± 4.81 | 22.08 ± 2.63 | 5.78 ± 0.65 | N.D. |
| **PE1** | 41.10 ± 6.29 | 30.50 ± 4.02 | 51.00 ± 5.09 | 31.80 ± 4.32 | 5.53 ± 0.94 | 4.36 ± 0.37 |
| **PE2** | 49.90 ± 7.72 | 30.30 ± 2.71 | 61.50 ± 5.23 | 28.50 ± 3.33 | 6.42 ± 0.52 | 3.80 ± 0.31 |
| **PE3** | 40.97 ± 5.93 | 29.64 ± 2.32 | 60.40 ± 6.58 | 24.10 ± 2.14 | 7.26 ± 0.64 | 5.21 ± 0.69 |
| **PE4** | 47.49 ± 6.22 | 28.53 ± 4.85 | 56.40 ± 7.53 | 32.57 ± 4.90 | 6.65 ± 1.11 | 4.55 ± 0.74 |
| **PE5** | 47.41 ± 6.75 | 29.72 ± 2.64 | 54.50 ± 4.59 | 30.87 ± 2.47 | 7.57 ± 0.78 | 4.13 ± 0.62 |
| **PE6** | 59.69 ± 5.15 | 28.43 ± 3.56 | 52.85 ± 7.31 | 35.84 ± 4.47 | 7.94 ± 0.96 | 5.42 ± 0.59 |
| **PE7** | 53.88 ± 6.83 | 39.55 ± 5.35 | 61.13 ± 4.67 | 26.80 ± 4.13 | 7.95 ± 1.19 | 4.57 ± 0.74 |
| **PE8** | 40.93 ± 6.35 | 29.06 ± 2.62 | 73.42 ± 12.48 | 28.88 ± 3.10 | 7.92 ± 0.79 | 3.83 ± 0.36 |
| **PE9** | 55.73 ± 7.90 | 39.50 ± 4.56 | 67.89 ± 11.38 | 26.24 ± 3.25 | 7.46 ± 0.90 | 5.38 ± 0.69 |
| **PS1** | 73.60 ± 10.85 | 29.90 ± 4.85 | 536.20 ± 92.11 | 40.90 ± 5.18 | 14.60 ± 1.51 | 15.90 ± 2.63 |
| **PS2** | 63.10 ± 5.67 | 31.18 ± 4.38 | 704.20 ± 105.80 | 59.67 ± 8.06 | 17.87 ± 2.73 | 16.50 ± 2.80 |
| **PS3** | 61.47 ± 6.76 | 28.24 ± 4.80 | 508.20 ± 49.54 | 65.81 ± 6.15 | 15.66 ± 2.08 | 16.10 ± 1.63 |
| **PS4** | 79.02 ± 10.62 | 32.71 ± 5.32 | 692.50 ± 105.19 | 51.75 ± 4.55 | 13.49 ± 2.07 | 15.80 ± 2.58 |
| **PS5** | 63.53 ± 6.54 | 30.31 ± 3.20 | 484.60 ± 46.71 | 49.45 ± 7.27 | 18.64 ± 3.16 | 20.20 ± 3.05 |
| **PS6** | 89.01 ± 10.59 | 32.69 ± 3.47 | 631.90 ± 87.23 | 49.85 ± 8.16 | 16.49 ± 2.01 | 18.90 ± 2.60 |
| **PS7** | 72.88 ± 9.86 | 31.99 ± 4.13 | 495.30 ± 40.17 | 65.79 ± 6.65 | 13.47 ± 2.20 | 21.10 ± 2.43 |
| **PS8** | 67.76 ± 5.23 | 36.83 ± 3.49 | 548.10 ± 81.19 | 60.20 ± 8.86 | 14.24 ± 2.41 | 15.30 ± 2.49 |
| **PS9** | 83.45 ± 8.80 | 27.86 ± 4.63 | 492.60 ± 52.73 | 46.53 ± 3.59 | 13.07 ± 1.05 | 15.40 ± 2.64 |
| **PVC1** | 278.45 ± 30.75 | 381.17 ± 34.32 | 182.20 ± 29.30 | 321.18 ± 39.81 | 79.10 ± 8.84 | 6.65 ± 0.76 |
| **PVC2** | 287.20 ± 45.77 | 472.04 ± 69.23 | 175.69 ± 22.54 | 318.27 ± 48.84 | 71.63 ± 8.00 | 7.56 ± 0.89 |
| **PVC3** | 264.90 ± 32.98 | 355.57 ± 52.30 | 238.27 ± 33.51 | 335.21 ± 44.29 | 89.74 ± 11.40 | 6.17 ± 0.62 |
| **PVC4** | 260.34 ± 32.37 | 429.02 ± 33.94 | 170.69 ± 24.48 | 323.93 ± 46.42 | 104.57 ± 12.06 | 5.77 ± 0.74 |
| **PVC5** | 332.97 ± 54.50 | 469.15 ± 51.22 | 198.73 ± 24.98 | 304.05 ± 25.66 | 70.79 ± 6.73 | 8.68 ± 0.90 |
| **PVC6** | 340.64 ± 41.63 | 496.56 ± 75.56 | 165.35 ± 14.14 | 366.08 ± 51.28 | 75.82 ± 7.33 | 6.11 ± 0.98 |
| **PVC7** | 357.69 ± 46.56 | 340.82 ± 37.59 | 239.82 ± 39.21 | 386.57 ± 39.88 | 91.35 ± 8.30 | 6.58 ± 0.55 |
| **PVC8** | 355.64 ± 54.19 | 505.45 ± 56.91 | 203.41 ± 20.11 | 305.16 ± 49.59 | 73.38 ± 8.90 | 8.20 ± 0.70 |
| **PVC9** | 288.66 ± 34.84 | 343.28 ± 29.75 | 201.15 ± 27.08 | 400.00 ± 56.56 | 80.69 ± 10.99 | 8.97 ± 0.81 |
| **Sample** | **Concentration (Mean ± SD, µg/kg of lipid)** | | | | |  |
|  | **Benzo[a]pyrene** | **Indeno[1,2,3-cd]pyrene** | **Dibenz[a,h]anthracene** | **Benzo[g,h,i]perylene** | **Total PAHs** |  |
| **W1** | 1.30 ± 0.18 | N.D. | 2.60 ± 0.37 | 2.84 ± 0.25 | 491.14 ± 62.29 |  |
| **W2** | 2.33 ± 0.35 | N.D. | 3.40 ± 0.37 | 4.15 ± 0.35 | 481.08 ± 58.09 |  |
| **W3** | 2.26 ± 0.27 | N.D. | 3.81 ± 0.47 | 4.55 ± 0.53 | 568.21 ± 70.49 |  |
| **W4** | 1.97 ± 0.23 | N.D. | 4.36 ± 0.73 | 4.06 ± 0.38 | 529.96 ± 89.71 |  |
| **W5** | 1.72 ± 0.17 | N.D. | 4.97 ± 0.79 | 4.08 ± 0.54 | 574.51 ± 76.15 |  |
| **W6** | 1.87 ± 0.18 | N.D. | 4.74 ± 0.78 | 4.58 ± 0.58 | 565.29 ± 81.29 |  |
| **W7** | 1.64 ± 0.28 | N.D. | 3.61 ± 0.48 | 3.24 ± 0.46 | 513.95 ± 83.05 |  |
| **W8** | 1.97 ± 0.16 | N.D. | 4.03 ± 0.38 | 4.23 ± 0.42 | 554.04 ± 78.95 |  |
| **W9** | 1.67 ± 0.24 | N.D. | 4.37 ± 0.60 | 4.13 ± 0.40 | 616.91 ± 69.78 |  |
| **PE1** | 2.84 ± 0.22 | 4.68 ± 0.61 | 7.25 ± 0.76 | 7.48 ± 1.28 | 1566.79 ± 160.59 |  |
| **PE2** | 2.66 ± 0.28 | 7.95 ± 1.27 | 7.66 ± 0.99 | 6.21 ± 0.91 | 1799.56 ± 197.45 |  |
| **PE3** | 3.17 ± 0.35 | 6.73 ± 1.12 | 9.15 ± 1.23 | 6.21 ± 0.90 | 1719.70 ± 186.09 |  |
| **PE4** | 2.89 ± 0.24 | 7.14 ± 1.12 | 7.32 ± 0.82 | 5.45 ± 0.79 | 1726.49 ± 280.43 |  |
| **PE5** | 2.93 ± 0.31 | 5.79 ± 0.48 | 7.73 ± 1.02 | 6.25 ± 1.02 | 1812.66 ± 285.73 |  |
| **PE6** | 3.15 ± 0.49 | 5.78 ± 0.96 | 9.30 ± 0.79 | 7.53 ± 0.72 | 1837.61 ± 276.90 |  |
| **PE7** | 3.32 ± 0.38 | 6.20 ± 0.67 | 8.39 ± 0.70 | 6.61 ± 1.02 | 1673.85 ± 261.40 |  |
| **PE8** | 2.59 ± 0.32 | 8.35 ± 1.01 | 9.06 ± 1.23 | 7.38 ± 0.74 | 1620.27 ± 220.72 |  |
| **PE9** | 2.86 ± 0.49 | 8.61 ± 1.01 | 4.06 ± 0.43 | 6.26 ± 0.95 | 1810.07 ± 172.35 |  |
| **PS1** | 3.39 ± 0.29 | 16.80 ± 2.22 | 25.30 ± 2.39 | 22.60 ± 2.93 | 1687.38 ± 196.64 |  |
| **PS2** | 3.23 ± 0.25 | 8.03 ± 0.73 | 25.90 ± 3.48 | 28.79 ± 3.78 | 1852.65 ± 283.91 |  |
| **PS3** | 3.37 ± 0.40 | 6.35 ± 0.59 | 23.20 ± 1.78 | 28.17 ± 2.27 | 1734.22 ± 183.22 |  |
| **PS4** | 4.20 ± 0.50 | 10.00 ± 0.97 | 30.00 ± 3.83 | 26.04 ± 3.63 | 1996.67 ± 323.36 |  |
| **PS5** | 3.00 ± 0.38 | 9.70 ± 1.01 | 24.50 ± 2.35 | 24.04 ± 3.64 | 1871.93 ± 292.43 |  |
| **PS6** | 3.10 ± 0.24 | 12.90 ± 1.50 | 32.50 ± 2.85 | 23.88 ± 2.03 | 1949.93 ± 168.43 |  |
| **PS7** | 4.20 ± 0.48 | 12.00 ± 1.33 | 24.60 ± 3.98 | 29.41 ± 4.04 | 1736.53 ± 139.00 |  |
| **PS8** | 3.20 ± 0.51 | 12.90 ± 1.59 | 29.70 ± 4.82 | 27.23 ± 4.24 | 1926.30 ± 230.83 |  |
| **PS9** | 4.40 ± 0.46 | 9.50 ± 1.13 | 28.20 ± 3.75 | 29.94 ± 4.53 | 1669.78 ± 133.35 |  |
| **PVC1** | 5.96 ± 0.49 | 71.10 ± 6.97 | 27.10 ± 3.79 | 48.10 ± 7.16 | 2523.99 ± 244.22 |  |
| **PVC2** | 5.91 ± 0.66 | 80.92 ± 7.44 | 32.92 ± 4.13 | 50.66 ± 8.47 | 2839.25 ± 475.74 |  |
| **PVC3** | 6.80 ± 0.53 | 66.84 ± 9.92 | 34.21 ± 2.90 | 53.00 ± 4.12 | 2692.14 ± 286.97 |  |
| **PVC4** | 5.57 ± 0.51 | 76.75 ± 7.82 | 29.99 ± 2.64 | 57.86 ± 8.61 | 2571.82 ± 259.22 |  |
| **PVC5** | 6.18 ± 0.54 | 83.05 ± 9.10 | 27.40 ± 3.66 | 50.16 ± 7.50 | 2915.44 ± 431.24 |  |
| **PVC6** | 7.86 ± 0.77 | 88.23 ± 14.99 | 28.16 ± 4.59 | 43.61 ± 4.73 | 2827.90 ± 218.74 |  |
| **PVC7** | 6.43 ± 1.10 | 93.30 ± 14.18 | 34.55 ± 2.81 | 45.71 ± 6.46 | 2894.12 ± 470.27 |  |
| **PVC8** | 7.04 ± 0.75 | 70.66 ± 9.98 | 25.42 ± 3.74 | 61.01 ± 6.30 | 2967.84 ± 366.09 |  |
| **PVC9** | 6.20 ± 0.58 | 67.91 ± 7.38 | 33.79 ± 4.08 | 61.40 ± 7.66 | 2664.73 ± 422.15 |  |

N.D.: not detected. W: wood; PE: wood + polyethylene; PS: wood + polystyrene; PVC: wood + polyvinyl chloride.
