# Supplementary material for: Effect of plastic composition in the combustion material on the Persistent Organic Pollutant content in smoked chicken meat
Source: PLoS One. 2026 Jun 3;21(6):e0350345. doi: 10.1371/journal.pone.0350345 (PMC13232828; doi:10.1371/journal.pone.0350345)
Supplement: S4 Table — Statistical significance was defined at p < 0.05, indicating that the corresponding PAH congener exhibited significant differences in concentration between the compared fuel groups. Values in bold indicate statistically significant differences. (DOCX) [file pone.0350345.s007.docx]

**Table S4. Pairwise comparisons were performed using the Wilcoxon signed-rank test with Benjamini–Hochberg correction for polycyclic aromatic hydrocarbons (PAHs) in smoked chicken meat samples across different fuel types (W, PE, PS, PVC). Statistical significance was defined at p < 0.05, indicating that the corresponding PAH congener exhibited significant differences in concentration between the compared fuel groups. Values in bold indicate statistically significant differences.**

| **Group1** | **Group2** | **Acenaphthene** | **Acenaphthylene** | **Benzo[a]anthracene** | **Benzo[a]pyrene** | **Benzo[b]fluoranthene** | **Benzo[e]pyrene** | **Benzo[g,h,i]perylene** | **Benzo[k]fluoranthene** |
| --- | --- | --- | --- | --- | --- | --- | --- | --- | --- |
| W | PE | **0.00**9 | **0.01**1 | **0.01**1 | **0.01**1 | **0.01**8 | **0.00**9 | **0.00**9 | **0.02**4 |
|  | PS | **0.009** | **0.011** | **0.011** | **0.011** | **0.011** | **0.009** | **0.009** | **0.011** |
|  | PVC | **0.009** | **0.011** | **0.011** | **0.011** | **0.011** | **0.009** | **0.009** | **0.011** |
| PE | PS | **0.00**9 | **0.01**1 | 1 | **0.01**3 | **0.01**1 | **0.00**9 | **0.00**9 | **0.01**1 |
|  | PVC | **0.009** | **0.011** | **0.011** | **0.011** | **0.011** | **0.009** | **0.009** | **0.011** |
| PS | PVC | **0.009** | **0.018** | **0.011** | **0.011** | **0.011** | **0.009** | **0.009** | **0.011** |

| **Group1** | **Group2** | **Chrysene** | **Dibenz[a,h]anthracene** | **Fluoranthene** | **Fluorene** | **Indeno[1,2,3-cd]pyrene** | **Naphthalene** | **Phenanthrene** | **Pyrene** |
| --- | --- | --- | --- | --- | --- | --- | --- | --- | --- |
| W | PE | **0.00**9 | **0.01**5 | **0.00**9 | **0.00**9 | **0.01**1 | **0.01**1 | **0.01**4 | **0.00**9 |
|  | PS | **0.009** | **0.014** | **0.009** | **0.009** | **0.011** | **0.018** | **0.014** | **0.009** |
|  | PVC | **0.009** | **0.014** | **0.009** | **0.009** | **0.011** | **0.011** | **0.014** | **0.009** |
| PE | PS | **0.00**9 | **0.01**4 | **0.00**9 | **0.00**9 | **0.01**8 | **0.01**1 | **0.01**5 | **0.00**9 |
|  | PVC | **0.009** | **0.014** | **0.009** | **0.009** | **0.011** | **0.011** | **0.014** | **0.009** |
| PS | PVC | **0.009** | 0.155 | **0.009** | **0.009** | **0.011** | **0.011** | 0.193 | **0.009** |
